# Selection and validation of suitable reference genes for qPCR gene expression analysis in goats and sheep under *Peste des petits ruminants virus (PPRV)*, lineage IV infection

**DOI:** 10.1038/s41598-018-34236-7

**Published:** 2018-10-29

**Authors:** Amit Ranjan Sahu, Sajad Ahmad Wani, Shikha Saxena, Kaushal Kishor Rajak, Dheeraj Chaudhary, Aditya Prasad Sahoo, Alok Khanduri, Aruna Pandey, Piyali Mondal, Waseem Akram Malla, Raja Ishaq Nabi Khan, Ashok Kumar Tiwari, Bina Mishra, D. Muthuchelvan, Bishnu Prasad Mishra, Raj Kumar Singh, Ravi Kumar Gandham

**Affiliations:** 1 Division of Veterinary Biotechnology, ICAR-IVRI, Izatnagar, Bareilly, UP 243122 India; 2 DBT-National Institute of Animal Biotechnology, Hyderabad, 500075 India; 3 The Ohio State University, Columbus, Ohio 43210 USA; 4 Division of Biological Products, ICAR-IVRI, Izatnagar, Bareilly, UP 243122 India; 5 Division of Virology, ICAR-IVRI, Mukteshwar Campus, Nainital, 263138 India; 6 ICAR-Directorate of Foot and Mouth Disease, Mukteshwar, Nainital, 263138 India; 7 Division of Biological Standardization, ICAR-IVRI, Izatnagar, Bareilly, UP 243122 India

## Abstract

Identification of suitable candidate reference genes is an important prerequisite for validating the gene expression data obtained from downstream analysis of RNA sequencing using quantitative real time PCR (qRT-PCR). Though existence of a universal reference gene is myth, commonly used reference genes can be assessed for expression stability to confer their suitability to be used as candidate reference genes in gene expression studies. In this study, we evaluated the expression stability of ten most commonly used reference genes (*GAPDH*, *ACTB*, *HSP90*, *HMBS*, *18S rRNA*, *B2M*, *POLR2A*, *HPRT1*, *ACAC*, *YWHAZ*) in fourteen different Peste des petits ruminants virus (PPRV) infected tissues of goats and sheep. RefFinder and RankAggreg software were used to deduce comprehensive ranking of reference genes. Our results suggested *HMBS* and *B2M* in goats and *HMBS* and *HPRT1* in sheep can be used as suitable endogenous controls in gene expression studies of PPRV infection irrespective of tissues and condition as a whole, thus eliminating the use of tissue specific/ condition specific endogenous controls. We report for the first time suitable reference genes for gene expression studies in PPRV infected tissues. The reference genes determined here can be useful for future studies on gene expression in sheep and goat infected with PPRV, thus saving extra efforts and time of repeating the reference gene determination and validation.

## Introduction

In the era of high throughput sequencing, RNA–Sequencing (RNA-Seq) has been widely applied to evaluate global gene expression levels and composition^1–3^. RNA-Seq produces reproducible results with little technical variation due to its immense power^4,5^. It offers a clear-cut measure of gene expression over a wide dynamic range^5,6^. Validation is an important part in a RNA-Seq experiment^7^. The differentially expressed genes identified are often validated using quantitative RT-PCR (qRT-PCR)^8,9^.

qRT-PCR is the premier molecular biological technique to define accurate expression profiles of selected genes of interest^10,11^. It is highly sensitive, specific and reproducible^12,13^ and acts as a key factor in the systems biology based studies where both quality control and validation are essential criteria^11^. The major concern in qRT-PCR is a suitable endogenous control/reference gene to nullify variations that arise in the due course of experiment^14^. The variation can be introduced at any step starting from RNA extraction to quantification of qRT-PCR in terms of quality and quantity^15,16^. Endogenous control genes are assumed to be constitutively and uniformly expressed within the samples to be compared, irrespective of experimental conditions or treatments and tissue differentiation^14,17^. Housekeeping genes are the most commonly used endogenous control genes. These genes are used as reference control genes to normalize the variations in the qRT-PCR experiment^18,19^. However, varying expression of housekeeping genes under different experimental conditions has been reported in viral infections^10,20–27^, cancer research^14,21^ and heat stress response in sheep^22^. The use of an invalidated reference gene in normalization leads to unreliable conclusions especially when used with tissue samples^15,18,23^. This warrants for a need to identify suitable reference gene(s) for normalization for every gene expression experiment to do away with the hurdles in qRT-PCR^24^. Sometimes, validated endogenous controls for the desired experimental conditions can be derived from the literature describing the similar type of experiment.

Peste des petits ruminants (PPR) is one of the most economically important diseases of goats and sheep, characterized by acute febrile condition, erosive stomatitis, diarrhea and pneumonia^25–27^. Eradication of rinderpest (RP) has put PPR in spotlight to be the next eradicable disease due to similar nature of the causative agent^28,29^. PPR caused by Peste des petits ruminants virus (PPRV) belongs to genus *Morbillivirus* of family *Paramyxoviridae*. It is known that virus infection (e.g. SARS corona virus, yellow fever virus, human herpes virus, cytomegalovirus etc.) often results in modified or fluctuating gene transcription patterns of conventionally used housekeeping genes^30,31^. Recently, *GAPDH* was identified to be the most suitable reference gene for evaluating the gene expression in PPRV infected goats PBMCs *in vitro*^9^. No published data is currently available on the use of specific reference gene(s) in goats and sheep infected with PPRV *in vivo*. RNA-Seq experiments are being carried out in our laboratory to identify specific host gene expressions signatures in goats and sheep under PPRV infection. The indiscriminate use of any endogenous control to validate the RNA–Seq experiment may lead to erroneous conclusions. Therefore, in our study we used a panel of ten reference genes viz. *GAPDH* (Glyceraldehyde-3-phosphate dehydrogenase), *18S rRNA* (18S ribosomal RNA), *B2M* (β 2 microglobulin), *HSP 90* (heat shock protein 90), *ACAC-alpha* (Acetyl coenzyme carboxylase alpha), *HMBS* (Hydroxymethylbilane synthase), *YWHAZ* (Tyrosine 3-monooxygenase activation protein zeta polypeptide), *POLR2A* (Polymerase^32^ II (DNA directed) polypeptide A), *ACTB* (beta actin) and *HPRT1* (Hypoxanthin Phosphoribosyl transferase 1) in fourteen different tissues obtained from healthy and PPRV infected goats and sheep to identify the best possible reference gene(s) for qRT-PCR normalization. We recommend different sets of reference genes based on the experimental condition.

## Results

### Performance of qRT-PCR primers

Gene specific amplification was confirmed by a single peak in the melting-curve analysis for all the genes (Supplementary Figs S1 and S2). The linear regression equation, correlation coefficient, PCR efficiency and standard curve for each gene are shown in Supplementary Figs S3 and S4. The efficiency of all the primers for the genes ranged from 93–107%.

### Confirmation of viral infection in tissue samples

The tissue samples were found positive for PPRV by sandwich ELISA, N gene based RT-PCR and qRT-PCR (Supplementary Fig. S5) and histopathology and immunohistochemistry (data not shown). All tissue samples of both the species had OD value above the cut off indicating the presence of PPRV antigen (Supplementary Figs S5A and S5B). The standard curve generated for N gene had the efficiency of 100.29%, and R^2^ -0.968 with a slope of −3.315. Expression of PPRV - N gene was detected in all the infected tissues of goats and sheep by qRT-PCR (Supplementary Figs S5A and S5B). RT-PCR revealed positive N gene amplicon of 351 bp for all the tissues from infected animals (Supplementary Figs S5C and S5D).

### Ct value of candidate reference genes

The mean Ct value of the genes in control, infected and combined groups is given in Supplementary Table S1. The mean Ct value of the reference genes ranged from 26.939 ± 0.153 (*B2M*) to 28.938 ± 0.191 (*ACTB*) in goats and 26.339 ± 0.19 (*ACTB*) to 28.332 ± 0.166 (*POLR2A*) in goats. Expression profile of all the 10 reference genes in both the species is represented by box whisker plots (Supplementary Fig. S6).

### Stability of candidate reference genes under specific experimental conditions

The lower the M-value coefficient, higher is the stability ranking in geNorm and NormFinder. In control goats, infected goats and goats combined, *B2M* and *HSP90*; *GAPDH* and *HMBS;* and *ACAC* and *HMBS*, respectively, were the most stable candidate genes by geNorm analysis. Similarly, *HMBS* and *HPRT1* were co-ranked as most stable genes in control sheep, infected sheep and sheep combined group by geNorm analysis (Table 1 and Supplementary Table S2). NormFinder and comparative delta Ct method analysis ranked *HMBS* as the stable gene for control goats, infected goats, goats combined, control sheep and sheep combined groups, and *HSP90* for infected sheep group (Table 1 and Supplementary Tables S3 and S4). The stability of a gene is inversely proportional to the standard deviation value in the BestKeeper algorithm. *HMBS* was found to be most stable reference gene in all groups of goats as well as for infected sheep and sheep combined groups and *B2M* for control sheep group (Table 1 and Supplementary Table S5).

**Table 1:** List of most stable endogenous control genes as suggested by different algorithms.

Group	geNorm	NormFinder	BestKeeper	Comparative delta Ct	RefFinder	RankAggreg
Control Goats	*B2M*, *HSP90*	*HMBS*	*HMBS*	*HMBS*	*HMBS*	*HMBS*
Infected Goats	*GAPDH*, *HMBS*	*HMBS*	*HMBS*	*HMBS*	*HMBS*	*HMBS*
Goats Combined	*ACAC*, *HMBS*	*HMBS*	*HMBS*	*HMBS*	*HMBS*	*HMBS*
Control Sheep	*HMBS*, *HPRT1*	*HMBS*	*B2M*	*HMBS*	*HMBS*	*HMBS*
Infected Sheep	*HMBS*, *HPRT1*	*HSP90*	*HMBS*	*HSP90*	*HSP90*	*HMBS*
Sheep Combined	*HMBS*, *HPRT1*	*HMBS*	*HMBS*	*HMBS*	*HMBS*	*HMBS*

### Comprehensive ranking of reference genes

**Figure 1:**
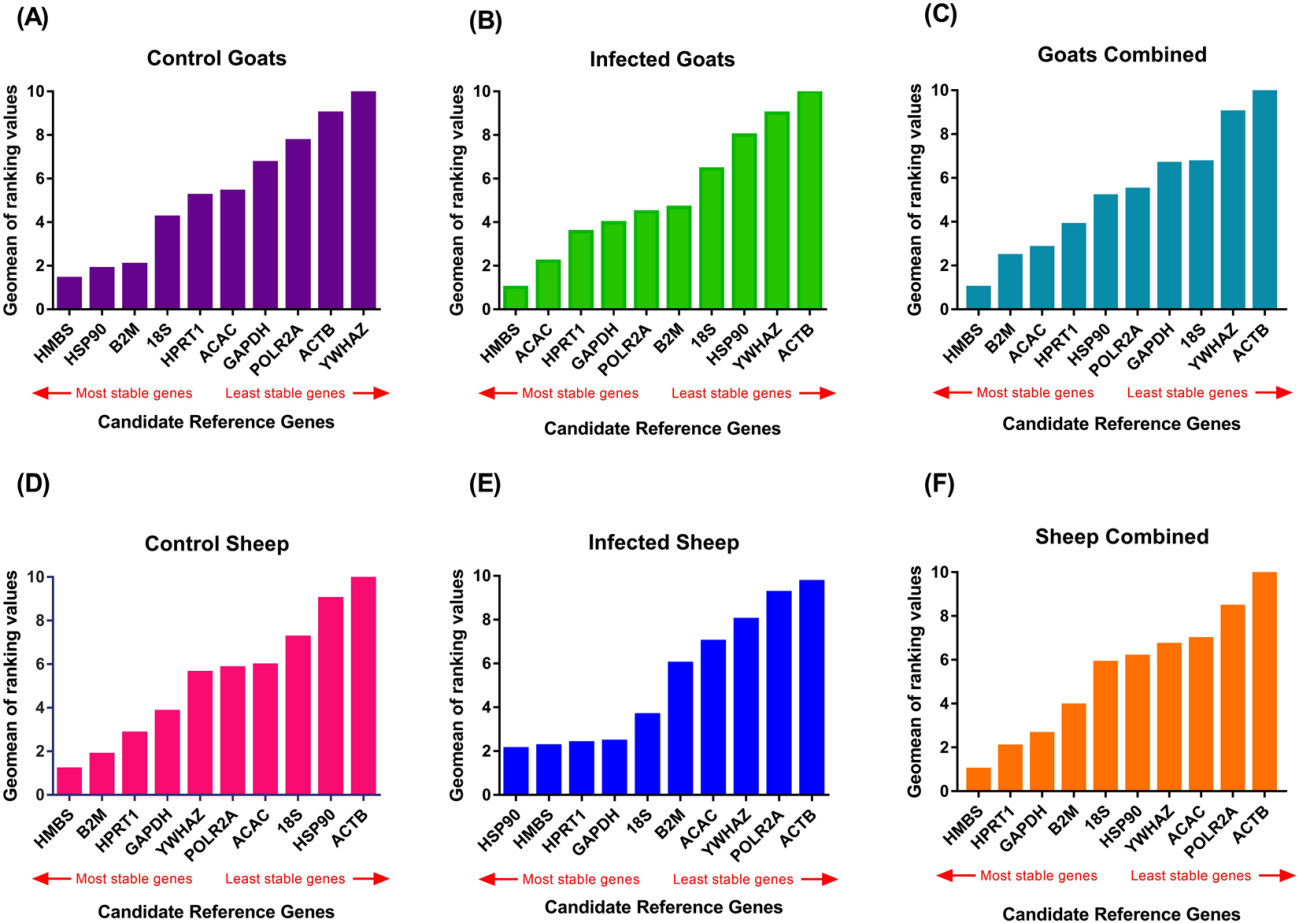
Comprehensive ranking pattern of ten candidate reference genes by RefFinder. (**A**) Control Goats (*HMBS*). (**B**) Infected Goats (*HMBS*). (**C**) Goats combined (*HMBS*). (**D**) Control Sheep (*HMBS*). (**E**) Infected Sheep (*HSP90*). (**F**) Sheep combined (*HMBS*).

**Figure 2:**
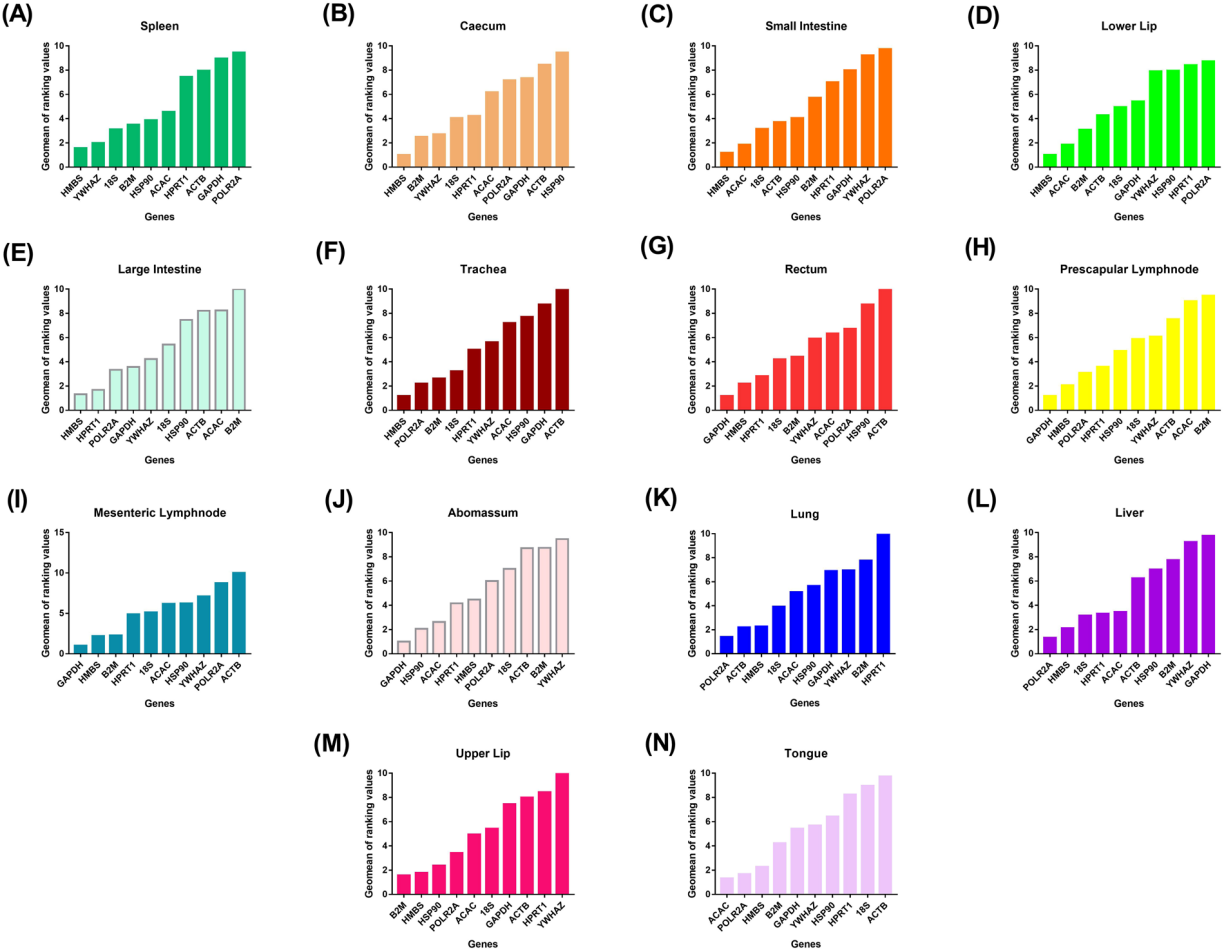
Comprehensive ranking pattern of ten candidate reference genes by RefFinder in fourteen different goat tissues. Most stable reference genes for each tissue are as follows- Spleen, Caecum, Small intestine, Lower lip, Large intestine, Trachea- *HMBS* (**A**–**F**); Rectum, Prescapular lymph node, Mesenteric lymph node, Abomasum- *GAPDH* (**G**–**J**); Lung and Liver- *POLR2A* (**K**,**L**); Upper lip- *B2M* (**M**); Tongue- *ACAC* (**N**).

**Figure 3:**
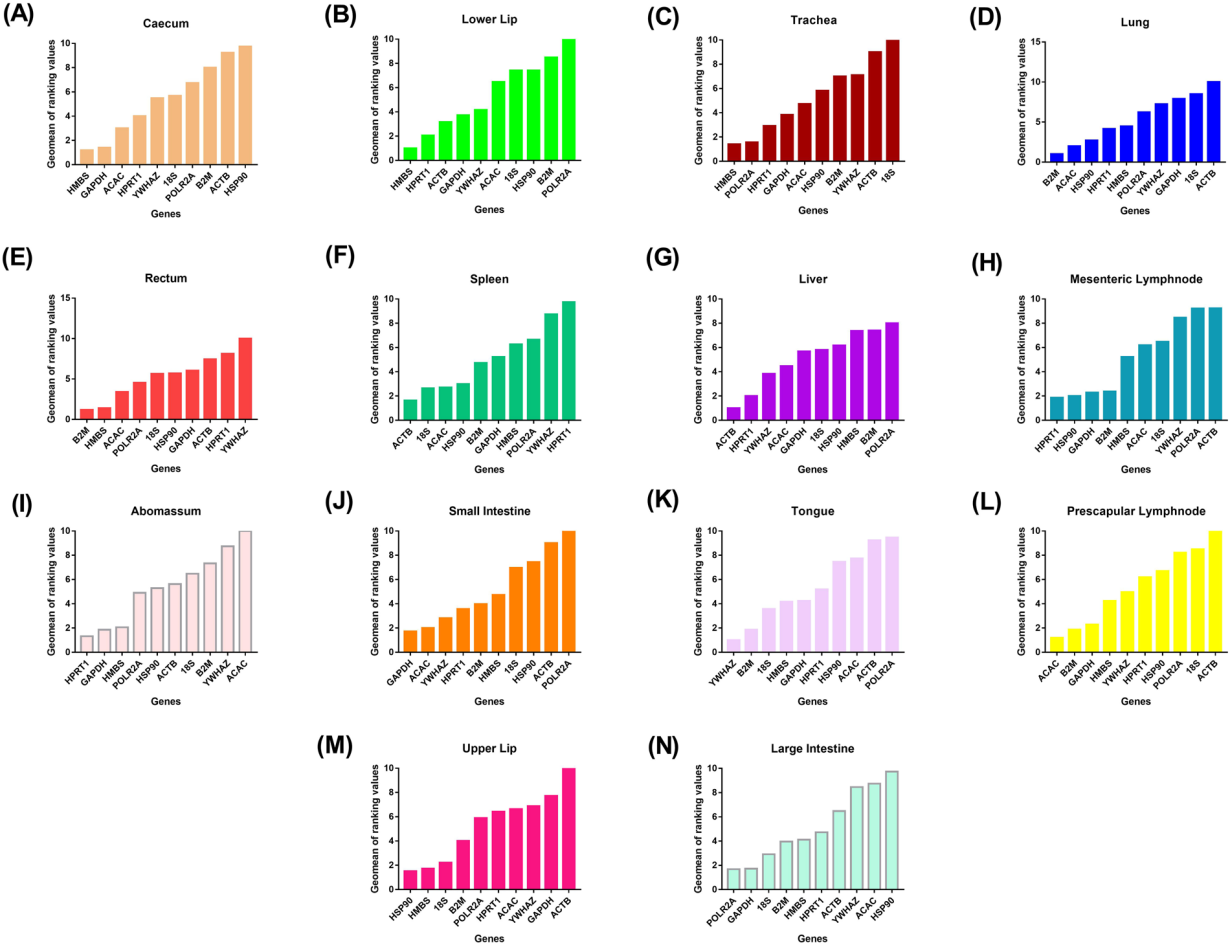
Comprehensive ranking pattern of ten candidate reference genes by RefFinder in fourteen different sheep tissues. Most stable reference genes for each tissue are as follows: Caecum, Lower lip, Trachea- *HMBS* (**A**–**C**); Lung, Rectum- *B2M* (**D**,**E**); Spleen, Liver- *ACTB* (**F**,**G**); Mesenteric lymph node, Abomasum- *HPRT1* (**H**,**I**); Small intestine- *GAPDH* (**J**); Tongue- *YWHAZ* (**K**); Prescapular lymph node- *ACAC* (**L**); Upper lip- *HSP90* (**M**); Large intestine- *POLR2A* (**N**).

RefFinder is a comprehensive program that integrates all four above mentioned software tools to rank the candidate reference genes based on their stability. The overall ranking suggested *HMBS* (Fig. 1A–F and Table 1 and Supplementary Table S6) to be the most stable among all groups of goats, control sheep and sheep combined groups while *HSP 90* (Fig. 1E) was found to be the most stable reference genes in infected sheep group. Tissue specific studies among various goat tissues revealed *HMBS* as the most stable gene in spleen (Fig. 2A), caecum (Fig. 2B), small intestine (Fig. 2C), lower lip (Fig. 2D), large intestine (Fig. 2E) and trachea (Fig. 2F); *GAPDH* in rectum (Fig. 2G), prescapular lymph node (Fig. 2H), mesenteric lymph node (Fig. 2I) and abomasum (Fig. 2J); *POLR2A* in lung (Fig. 2K) and liver (Fig. 2L); *B2M* in upper lip (Fig. 2M) and *ACAC* in tongue (Fig. 2N). Comprehensive ranking among tissue specific studies in sheep suggested *HMBS* as the most stable gene in caecum (Fig. 3A), lower lip (Fig. 3B) and trachea (Fig. 3C); *B2M*, in lung (Fig. 3D) and rectum (Fig. 3E); *ACTB* in spleen (Fig. 3F) and liver (Fig. 3G); *HPRT1* in mesenteric lymph node (Fig. 3H) and abomasum (Fig. 3I); *GAPDH* in small intestine (Fig. 3J); *YWHAZ* in tongue (Fig. 3K); *ACAC* in prescapular lymph node (Fig. 3L); *HSP90* in upper lip (Fig. 3M) and *POLR2A* in large intestine (Fig. 3N).

RankAggreg provides the consensus ranking by BruteAggreg function of the package. RankAggreg suggested *HMBS* to be the most stable endogenous control gene in all groups of goats and sheep (Table 1 and Supplementary Table S6). Similarly, for tissues specific studies, RankAggreg suggested *HMBS* for caecum, large intestine, lower lip, small intestine, spleen and trachea; *GAPDH* for abomasum, mesenteric lymph node, prescapular lymph node and rectum; *POLR2A* for liver and lung; *ACAC* for tongue and *B2M* for upper lip as the most stable candidate reference genes in goats (Supplementary Table S7). In sheep, RankAggreg suggested *HMBS* for caecum, lower lip and trachea; *HPRT1* for abomasum and mesenteric lymph node; *GAPDH* for large intestine and small intestine; *ACTB* for liver and spleen; *B2M* for lung and rectum; *ACAC* for prescapular lymph node; *YWHAZ* for tongue and *HSP90* for upper lip, as the most stable genes (Supplementary Table S8). Final consensus ranking was obtained for each condition and tissue from RankAggreg results. The consensus ranking was obtained for each condition and tissue by considering the results of both RefFinder and RankAggreg. A detailed list of recommended endogenous control genes for individual tissues is given in Table 2.

**Table 2:** Recommended list of two most stable endogenous controls to be used in different conditions and different tissues.

Conditions/Tissue Samples	Goat	Sheep
Control	*HMBS*, *HSP90*	*HMBS*, *B2M*
Infected	*HMBS*, *ACAC*	*HMBS*, *HSP90*
Combined	*HMBS*, *B2M*	*HMBS*, *HPRT1*
Lung	*POLR2A*, *ACTB*	*B2M*, *ACAC*
Spleen	*HMBS*, *YWHAZ*	*ACTB*, *18S*
Caecum	*HMBS*, *B2M*	*HMBS*, *GAPDH*
Rectum	*GAPDH*, *HMBS*	*B2M*, *HMBS*
Small Intestine	*HMBS*, *ACAC*	*GAPDH*, *ACAC*
Prescapular Lymph node	*GAPDH*, *HMBS*	*ACAC*, *B2M*
Mesenteric Lymph node	*GAPDH*, *HMBS*	*HPRT1*, *HSP90*
Liver	*POLR2A*, *HMBS*	*ACTB*, *HPRT1*
Upper Lip	*B2M*, *HMBS*	*HSP90*, *HMBS*
Lower Lip	*HMBS*, *ACAC*	*HMBS*, *HPRT1*
Abomasum	*GAPDH*, *HSP90*	*HPRT1*, *GAPDH*
Tongue	*ACAC*, *POLR2A*	*YWHAZ*, *B2M*
Large Intestine	*HMBS*, *HPRT1*	*GAPDH*, *POLR2A*
Trachea	*HMBS*, *POLR2A*	*HMBS*, *POLR2A*

### Validation of suitable endogenous control genes

**Figure 4:**
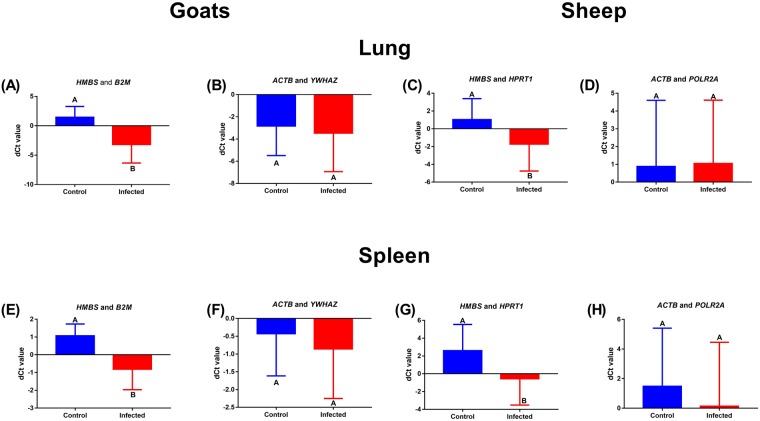
Expression of *ISG15* in lung and spleen tissues of both goats and sheep with two most stable reference genes (*HMBS* and *B2M* in goats; *HMBS* and *HPRT1* in sheep) and two least stable reference genes (*ACTB* and *YWHAZ* in goats; *ACTB* and *POLR2A* in sheep). *ISG15* expression in control and infected lung tissues of goats with the two most stable reference genes (**A**) and two least stable reference genes (**B**). *ISG15* expression in control and infected lung tissues of sheep with the two most stable reference genes (**C**) and two least stable reference genes (**D**). *ISG15* expression in control and infected spleen tissues of goats with two most stable reference gene (**E**) and two least stable reference genes (**F**). *ISG15* expression in control and infected spleen tissues of sheep with two most stable reference genes (**G**) and two least stable reference genes (**H**). The expression was calculated as delta Ct value (Ct_(*ISG15*)_ − Ct_(geometric mean of Ct of the best endogenous control genes)_ or Ct_(geometric mean of the least stable endogenous control genes)_). Significance (p < 0.05) of difference in expression between the control and infected groups was tested using t-test. Levels not connected by the same superscript are significantly (p < 0.05) different.

**Figure 5:**
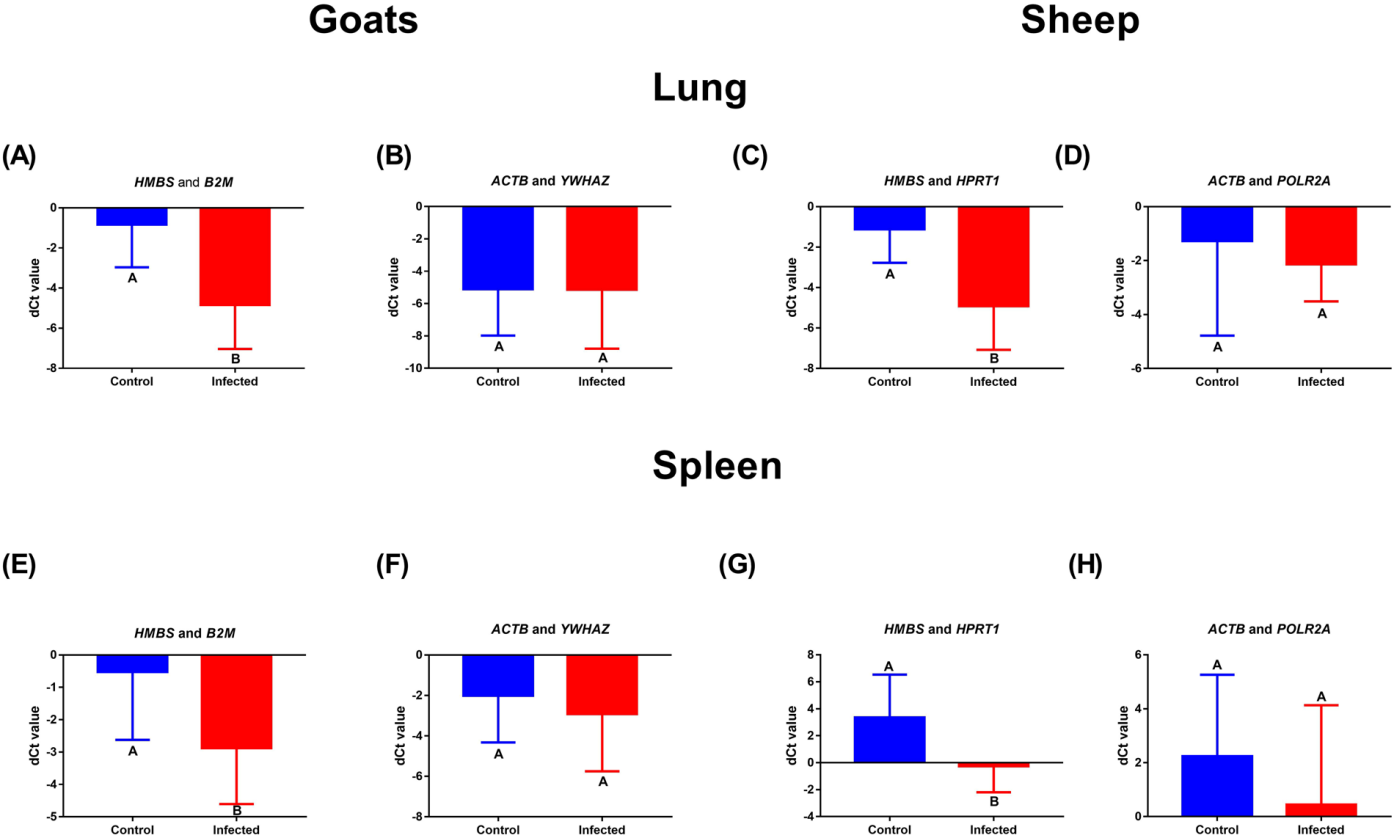
Expression of *IRF7* in lung and spleen tissues of both goats and sheep with two most stable reference genes (*HMBS* and *B2M* in goats; *HMBS* and *HPRT1* in sheep) and two least stable reference genes (*ACTB* and *YWHAZ* in goats; *ACTB* and *POLR2A* in sheep). *IRF7* expression in control and infected lung tissues of goats with the two most stable reference genes (**A**) and two least stable reference genes (**B**). *IRF7* expression in control and infected lung tissues of sheep with the two most stable reference genes (**C**) and two least stable reference genes (**D**). *IRF7* expression in control and infected spleen tissues of goats with two most stable reference gene (**E**) and two least stable reference genes (**F**). *IRF7* expression in control and infected spleen tissues of sheep with two most stable reference genes (**G**) and two least stable reference genes (**H**). The expression was calculated as delta Ct value (Ct_(*IRF7*)_ − Ct_(geometric mean of Ct of the best endogenous control genes)_ or Ct_(geometric mean of the least stable endogenous control genes)_). Significance (p < 0.05) of difference in expression between the control and infected groups was tested using t-test. Levels not connected by the same superscript are significantly (p < 0.05) different.

The amplification efficiency was 100.04% with R^2^ of 0.998 and slope of −3.3209 for *ISG15*, and amplification efficiency of 104.8%, R2 of 0.999 and slope of −3.2121 for *IRF7* (Supplementary Fig. S4). Significant (p ≤ 0.05) difference between the expression values (delta Ct values) of *ISG15* and *IRF7* in lung and spleen tissues of control and infected groups of both species was observed when the two best stable candidate reference genes were used as calibrator (Figs 4 and 5A,C,E,G), and no significant difference was obtained when two least stable endogenous controls were used as calibrator (Figs 4 and 5B,D,F,H).

## Discussion

Due to its high specificity and sensitivity, qRT-PCR dominated the world of gene expression studies among all other contemporary techniques. It is extremely useful in gene expression studies to document host cell responses to virus infection^9,20,31,33–41^. Elucidation of molecular pathogenesis from global gene expression profile by high-throughput omics study ultimately ends up in a number of candidate genes. qRT-PCR provides the simplest platform for its validation. In spite of these facts, qRT-PCR requires a robust normalization of the data to overcome the variability introduced at any of the steps in an experiment^42,43^. An ideal reference gene should be stably expressed in tissues under varied experimental conditions. However, this constant expression of any reference gene only refers to a specific condition under certain environment and the expression level in different cell types and tissues significantly varies under different experimental systems^41,44–46^. Thus identification and validation of reference genes for expression studies in an experiment is widely supported and practiced^23,47^.

The data in the study was analyzed using geNorm, NormFinder, BestKeeper and Comparative ΔCt method. geNorm calculates the standard deviation of the expression ratio of two candidate reference genes, which are not co-regulated as a pairwise variation^23^. The stability value (M) is calculated as the average pairwise variation of a specific gene compared with all other reference genes. Genes with the highest M values have the least stable expression. geNorm also identifies the number of reference genes required for the normalization of a particular experiment^48^. The NormFinder allows comparison of intra- and inter-group variation and calculates expression stability value (M)^49,50^. BestKeeper uses repeated pairwise correlation analysis to determine the optimal reference genes^51^. The comparative delta Ct method^52^ evaluates the average of standard deviation values derived from comparison of relative expression between a reference gene with other reference genes. The difference in ranking results obtained from different software programs in our study may be attributed to the use of different algorithms by different softwares to determine gene expression stability^41,43,53^. Most of the reports recommend consensus comprehensive ranking for use as best endogenous control^41,43,53^. Therefore, we recommend the candidate reference genes obtained through comprehensive ranking method in all the three different experimental conditions i.e. control, infected and combined. In control goats and control sheep, *HMBS* and *HSP90*, and *HMBS* and *B2M* are recommended as the most stable endogenous controls. These genes can be used as suitable reference genes in studies where basal expression of target genes across healthy tissues is compared in goats and sheep. In PPRV infected tissue studies for comparing across tissues, we recommend the use of *HMBS* and *ACAC* in goats and *HMBS* and *HSP90* in sheep. In comparative studies of PPRV infected with uninfected tissues as a whole, we recommend the use of *HMBS* and *B2M* in goats and *HMBS* and *HPRT1* in sheep, thus eliminating the use of multiple tissue specific endogenous controls. The purpose of the combined analysis was to demonstrate the stability of reference genes with respect to different conditions and tissues. We recommend to use that reference gene which shows highest stability in the combined groups for studies under PPRV infection.

ISG15 plays a key role in the innate immune response to viral infection either via its conjugation to a target protein (ISGylation) or via its action as a free or unconjugated protein. ISGylation involves a cascade of enzymatic reactions to alter host immune system. It exhibits antiviral activity towards both DNA and RNA viruses, including influenza A, HIV-1 and Ebola virus^27,54–56^. IRF7, a key innate immune modulator controlling the induction of type I interferons during viral infections^57,58^. Upon activation, phosphorylated IRF7 induce expression of genes responsible for type I interferon production inside the nucleus in virus infection^58^. *ISG15* and *IRF7* were chosen as the target gene of interest as these genes have been identified and predicted as important antiviral molecules by RNA–sequencing data analysis of PPRV infection studies in our lab (data not shown). The significant difference in expression of *ISG15* and *IRF7* on use of the most stable reference genes in goats and sheep corroborated with the findings of the RNA-Seq experiment conducted in the laboratory. The reference genes determined herein can be used by other researchers of the same field, thereby, saving the cost, effort and time of repeating the endogenous control determination and validation.

In conclusion, this study is the first attempt to establish suitable reference genes for gene expression studies in PPRV infection in fourteen different tissue samples of goats and sheep. Our results provide the starting point to validate candidate gene(s) expression obtained from transcriptome studies in PPRV infection.

## Materials and Methods

### Ethics Statement

The vaccine potency testing experiment was carried out at ICAR - Indian Veterinary Research Institute Mukteshwar Campus as per the guidelines of Indian Pharmacopia-2014. The study was done after obtaining permission from Indian Veterinary Research Institute Animal Ethics Committee (IVRI - IAEC) under the Committee for the Purpose of Control and Supervision of Experiments on Animals (CPCSEA), India. The protocols were approved vide letter no 387/CPCSEA. Apparently healthy, non-descriptive hill goats (local Rohilkhand breed) and sheep (Muzaffarnagri breed) between 6 months to 1 year of age were used in the present study. Virulent PPRV (Izatnagar/94- lineage IV, accession number KR140086.1)^59^ isolate was used as a challenge virus for infection. The tissue samples - upper lip, lower lip, tongue, trachea, lung, pre-scapular lymph node, mesenteric lymph node, spleen, liver, small intestine, large intestine, abomasum, caecum and rectum were collected from PPRV infected sheep and goats (n = 6 for each of the species). The counterpart healthy tissues were collected from apparently healthy animals (negative for PPRV antibody by competitive ELISA and serum neutralization test) housed separately. The apparently healthy animals are referred as control. These animals were handled in a humane manner and euthanized as per the CPCSEA guidelines. The graphical abstract of this study is represented in Supplementary Fig. S7.

### Selection of Candidate Reference genes

A total of ten candidate reference genes were selected based on, their use as reference genes in diverse studies on gene expression in goats and sheep, availability of their sequences in databases and their function in the cell (Table 3). The ten reference genes used were *GAPDH*, *ACTB*, *B2M*, *HSP 90*, *ACAC-α*, *HMBS*, *YWHAZ*, *POLR2A*, *HPRT1* and *18S rRNA*^9,31,35,38–41,43,53,60^. Primers for *YWHAZ*, *POLR2A*, *ACTB* and *HPRT1* were designed based on the sequence obtained from NCBI with the help of software Primer3Plus^61^. The quality parameters for these primers were checked in Oligo Analyzer and NCBI Primer BLAST^62^. The primers for rest of the genes were obtained from already published literature^9,41,53^.

**Table 3:** Selected candidate reference genes used in the qRT-PCR assay.

Gene Symbol	Gene Name	Function	Accession No.	Primer Sequence	Amplicon Size	Efficiency
*GAPDH*	Glyceraldehyde 3-phosphate dehydrogenase	Glycolytic enzyme, Oxidoreductase in glycolysis and gluconeogenesis	NM_001206359.1	FP: TGGTGAAGGTCGGAGTGAAC RP: GGAAGATGGTGATGGGATTTC	225 bp	95.73
*18S rRNA*	Eukaryotic 18S ribosomal RNA	Ribosomal RNA, Component of ribosomal protein	DQ149973.1	FP: TAATCCCGCCGAACCCCATT RP: GGTGTGTACAAAGGGCAGG	125 bp	97.36
*B2M*	β-2-microglobulin	Cell surface molecule component, MHC class l molecule	XM_012180604.1	FP: TGT CCC ACG CTG AGT TCA CT RP: TGA GGC ATC GTC AGA CCT TGA	137 bp	107.57
*HSP 90*	Heat Shock Protein 90 kDa	Protein Folding, Protein degradation	XM_004017995.3	FP: GCC CGA GAT AGA AGA CGT TG RP: AGT CGT TGG TCA GGC TCT TG	197 bp	95.49
*ACAC*	Acetyl coenzyme A carboxylase alpha (ACAC-α)	Regulate metabolism of fatty acid	NM_001009256	FP: CGC TAT GGA AGT CGG CTG TG RP: CAG GAA GAG GCG GAT GGG AA	105 bp	93.01
*HMBS*	Hydroxymethyl-bilane synthase	Heme biosynthesis	XM_012095569.2	FP: CTT GCC AGA GAA GAG TGT GG RP: CAG CCG TGT GTT GA GGT TTC	115 bp	97.54
*YWHAZ*	Tyrosine 3-monooxygenase activation protein zeta polypeptide	Signal transduction	NM_174794.2	FP: TTC TGA GGT GGC TTC TGG AG RP: AGT CGA ATG GGG TGT GTA GG	117 bp	96.93
*POLR2A*	Polymerase II (DNA directed) polypeptide A	DNA-dependent RNA polymerase	NM_001206313.1	FP: AGA GGT GGT GGA CAA GAT GG RP: ACA CCT TGC TGA TCT GCT CT	104 bp	94.76
*ACTB*	Beta-Actin	Cytoskeletal structural protein	NM_001314342.1	FP: CTC TTC CAG CCT TCC TTC CT RP: TAA AGG TCC TTG CGG ATG TC	101 bp	102.77
*HPRT1*	Hypoxanthine phosphoribosyltransferase 1	Purine synthesis in salvage pathway	XM_013976270.1	FP: CACTGGGAAGACAATGCAGA RP: ACACTTCGAGGGGTCCTTTT	102 bp	99.51

### RNA extraction and cDNA synthesis

About 30–50 mg tissues were cut into slices and dipped in TRIzol at 4 °C. The tissues were homogenized with tissue ruptor (QIAgen, Cat No: 9001271) and total RNA was isolated from the homogenized tissue using Ribozol™ RNA extraction reagent (Amresco LLC, Solon, USA) as per manufacturer’s protocol. Total RNA was dissolved in 30 μl ddH_2_O and quantified using NanoDrop 1000 Spectrophotometer (Thermo Fisher Scientific Inc., Wilmington, DE, USA). One microgram of total RNA from each sample was treated with DNAse I, RNAse free enzyme (Thermo Fisher Scientific Inc., Cat No. EN0521) in the presence of 40U of RNAse inhibitor (RNAseOUT, Invitrogen), following the instructions of the manufacturer. One microgram of treated RNA was reverse transcribed using a RevertAid™ First Strand cDNA Synthesis Kit (Thermo Fisher Scientific Inc., Wilmington, DE, USA, Cat No. K1622) according to the manufacturer’s protocol. Briefly, 12 μl of a mixture 1 was prepared taking 1000 ng of RNA along with 1 μl of random primers and volume adjusted by NFW (Nuclease free water, Himedia, Cat No. TCL018). Mixture 1 was incubated at 65 °C for 5 min and then snap chilled at 25 °C for 5 min. To this 12 μl of mixture 1, 8 μl of mixture 2 (4 μl of 5X RT buffer, 2 μl of 10 mM dNTPs, 1 μl each of Ribolock inhibitor and Reverse Transcriptase enzyme) was added and the final mixture of 20 μl was incubated initially at 25 °C for 10 min, then at 42 °C for 1 hour followed by 72 °C for 10 min and finally at 40 °C for 10 min. The cDNA thus obtained was stored at −20 °C till further use.

### Confirmation of PPRV Infection in Tissue Samples

#### Sandwich ELISA (sELISA) of the Tissue Samples

PPR sandwich-ELISA kit for PPRV antigen detection was obtained from National Morbilivirus referral laboratory, Division of Virology, IVRI, Mukteshwar^63^. sELISA for the tissue samples was carried out as per the instructions provided with the kit.

#### RT-PCR of N gene in Tissue samples

Diagnostic PCR for N gene was carried out with the help of published primers PPRV-NP3-FP 5′-TCTCGGAAATCGCCTCACAGACTG-3′ and PPRV NP4-RP 5′-CCTCCTCCTGGTCCTCCAGAATCT-3′^64^. 25 μl of reaction mixture was prepared with 2.5 μl of 10X DreamTaq buffer, 1 μl of 10 mM dNTPs (Thermo Fisher Scientific Inc., Wilmington, DE, USA, Cat No. R0181), 0.5 μl of 10 pM each of forward and reverse primers, 0.25 μl of 1.25 U DreamTaq DNA polymerase (Thermo Fisher Scientific Inc., Wilmington, DE, USA, Cat. No. EP0702), 2 μl of 100 ng template cDNA and 18.25 μl of NFW. PCR was carried out with an initial denaturation at 95 °C for 5 min followed by 35 cycles of denaturation at 95 °C for 30 sec, annealing at 60 °C for 30 sec, renaturation at 72 °C for 30 sec with a final extension step at 72 °C for 5 min. PCR product was visualized on 1.5% agarose gel.

#### Absolute quantification of N gene in all tissues

Absolute quantification of N gene for viral load by qRT-PCR for infected tissues was performed using primers specific to PPRV N gene. Primers – PPRV N-FP: ATCTGCAGGAAAGGTCAGCT-3′ and PPRV N-RP: TCCCTCTCCTGTTTTGTGCT-3′ were designed using Primer3plus. The standard curve was generated using a series of 10-fold dilutions of gel purified PCR product of N gene. The amplification efficiency was calculated from the slope of the standard curve using the formula E = 10^(−1/slope)^. Copy number was calculated from the standard curve (Supplementary Fig. S5). Ct values greater than 35 were considered negative.

#### Reverse transcriptase-quantitative polymerase chain reaction (qRT-PCR) of reference genes

Gene specific primers (Table 3) were used in PCR reactions of 10 µl containing 5 µl of 2X Maxima SYBR Green qPCR MasterMix (Thermo Fisher Scientific Inc., Wilmington, DE, USA, Cat. No. K0251), 0.1 µl of 10 pm/µl of forward and reverse primers, 0.1 µl of ROX dye and 2.7 µl of Nuclease free water with 10 ng (2 µl) of template cDNA. The reactions were set up in MicroAmp Fast 96 well reaction plate (0.1 ml) (Applied Biosystems) in triplicates. Reactions were performed in a 7500 Fast Real Time PCR detection system (Applied Biosystem, USA) for all the tissues of control (uninfected) and PPRV infected animals. The efficiency of Real-Time PCR and slope values were determined for each primer. The standard curve was generated using a series of 10 fold dilutions. The amplification efficiency was calculated from the slope of the standard curve using the formula E = 10^(−1/slope)^. A melting curve analysis confirmed the presence of a single gene specific peak and the absence of primer dimers. Melting curve analysis consisted of 95 °C for 15 sec, 60 °C for 1 min, followed by 95 °C for 30 sec and a final step of 60 °C for 15 sec.

#### Data analysis

The Ct values for the control (uninfected), and PPRV infected samples were initially analyzed for each of the species to determine the best possible endogenous control(s) for healthy and PPRV infected conditions separately. Then, the data (Ct values) from the control and infected were combined for each of the species to identify the best endogenous control for the case where PPRV infected samples were compared with control healthy samples. The data was analyzed for six groups: Goats – control goats, infected goats (PPRV infected) and goats combined (combining both the control and infected Ct values); Sheep – control sheep, infected sheep and sheep combined. To determine tissue specific endogenous controls, both infected and control tissue Ct values were taken into consideration for each species. Stability of the 10 candidate reference genes were evaluated by algorithms geNorm^23^, NormFinder^49^, BestKeeper^51^, and the comparative Δ delta Ct method^52^ in RefFinder (http://leonxie.esy.es/RefFinder/). A comprehensive overall ranking of the stability by integrating all four algorithms was provided in the RefFinder.

The final consensus ranking was obtained with RankAggreg package^65^ by considering the results obtained from all the above analysis. The RankAggreg package of R software combines the stability measurements obtained from softwares (geNorm, NormFinder, BestKeeper, comparative delta Ct method and RefFinder) and establishes a consensus rank of reference genes^65^. A weighted rank aggregation was applied by using BruteAggreg function of the package. This function performs rank aggregation using the brute force approach. The aim of rank aggregation is to find an aggregated ranking that minimizes the distance to each of the ranked lists in the input set.

#### Validation of reference genes identified

The stability of the identified best reference genes was validated by evaluating the expression of *ISG15* and *IRF7* as target genes in the PPRV infected lung and spleen tissues with respect to the control tissues in both the species. *ISG15* and *IRF7* were chosen as the target genes of interest as these genes have been identified and predicted as important antiviral molecules by RNA–sequencing data analysis of PPRV infection studies in our lab. Forward primer 5′-CAGTTCATCGCCCAGAAGAT-3′ and reverse primer 5′-GTCGTTCCTCACCAGG ATGT-3′ were used for *ISG15*. Similarly, for *IRF7* 5′-GACACGCCCATCTTTGACTT-3′ and 5′- ACTGTCCAGGGAGGACACAC-3′ were used as primers. The amplification efficiency was calculated from the standard curve generated by 7 point, 10 fold serial dilutions. The Ct values for control and infected tissue samples with two most stable endogenous control genes (*HMBS* and *B2M* in goats, and *HMBS* and *HPRT1* in sheep) and two least stable endogenous control gene (*ACTB* and *YWHAZ* for goats and *ACTB* and *POLR2A* for sheep) were estimated. Expression for control and infected groups was represented by delta Ct value (Ct_(target genes)_ − Ct_(geometric mean of Ct of the two best endogenous control genes)_ or Ct_(geometric mean of Ct of two least stable endogenous control genes)_). t- test in GraphPad Prism 7 was used to compare the expression of *ISG15* and *IRF7* in infected relative to control.

#### MIQE guidelines

This enables the researcher to evaluate the technical quality of the qRT-PCR experiments^66,67^. All the experiments were carried out as per the MIQE guidelines. A summary sheet of MIQE guideline of this experiment is provided in Supplementary Table S9.

## Electronic supplementary material

Supplementary Information
