# Supplementary material for: Selection and validation of suitable reference genes for qPCR gene expression analysis in goats and sheep under Peste des petits ruminants virus (PPRV), lineage IV infection
Source: Sci Rep. 2018 Oct 29;8:15969. doi: 10.1038/s41598-018-34236-7 (PMC6206032; doi:10.1038/s41598-018-34236-7)
Supplement: Supplementary file 1 — Supplementary Information [file 41598_2018_34236_MOESM1_ESM.pdf]

Selection and validation of suitable reference genes for qPCR gene expression analysis in goats and sheep under *Peste des petits ruminants virus (PPRV)*, lineage IV infection

Amit Ranjan Sahu, Sajad Ahmad Wani, Shikha Saxena, Kaushal Kishor Rajak, Dheeraj Chaudhary, Aditya Prasad Sahoo, Alok Khanduri, Aruna Pandey, Piyali Mondal, Waseem Akram Malla, Raja Ishaq Nabi Khan, Ashok Kumar Tiwari, Bina Mishra, D. Muthuchelvan, Bishnu Prasad Mishra, Raj Kumar Singh, Ravi Kumar Gandham

Supplementary Figure Legends

Supplementary Figure S1: Melting curve of ten candidate reference genes.

Supplementary Figure S2: Melting curve of *PPRV N* gene, *ISG15* and *IRF7*.

Supplementary Figure S3: Standard curve, slope, R^2, efficiency for all the ten candidate reference genes.

Supplementary Figure S4: Standard curve, slope, R^2, efficiency for *PPRV N* gene, *ISG15* and *IRF7*.

Supplementary Figure S5: Representative figure for confirmation of viral infection in tissues. (A) qRT-PCR and sELISA in different tissues of goats. qRT-PCR N gene copy number (primary axis) is indicated in red and sELISA absorbance (secondary axis) is indicated in blue. (B) qRT-PCR and sELISA in different tissues of sheep. qRT-PCR N gene copy number (primary axis) is indicated in red and sELISA absorbance (secondary axis) is indicated in blue. (C) Amplification of 351bp N gene specific fragment by RT-PCR in tissue samples of goat. Gel 1: M- Marker, 1- NTC, 2- Caecum, 3- Lung, 4- Spleen, 5- Rectum, 6- Small Intestine, 7- Liver; Gel 2: M- Marker, 1- NTC, 2- Lower lip, 3- Abomassum, 4- Large Intestine, 5- Prescapular lymphnode, 6- Mesenteric lymphnode, 7-Trachea; Gel 3: M- Marker, 1- NTC, 2- Upper lip, 3- Tongue. (D) Amplification of 351bp N gene specific fragment by RT-PCR in tissue samples of sheep. Gel 1: M- Marker, 1- NTC, 2- Caecum, 3- Lung, 4- Spleen, 5- Rectum, 6- Small Intestine, 7- Prescapular lymphnode; Gel 2: M- Marker, 1- NTC, 2- Mesenteric lymphnode, 3- Liver, 4- Upper lip, 5- Lower lip, 6- Tongue, 7- Large Intestine; Gel 3: M- Marker, 1- NTC, 2- Abomassum, 3- Trachea.

Supplementary Figure S6: Expression profile of ten candidate reference genes. Box-Whisker plot for Ct values in Goats and Sheep.

Supplementary Figure S7: Graphical abstract depicting the current study.

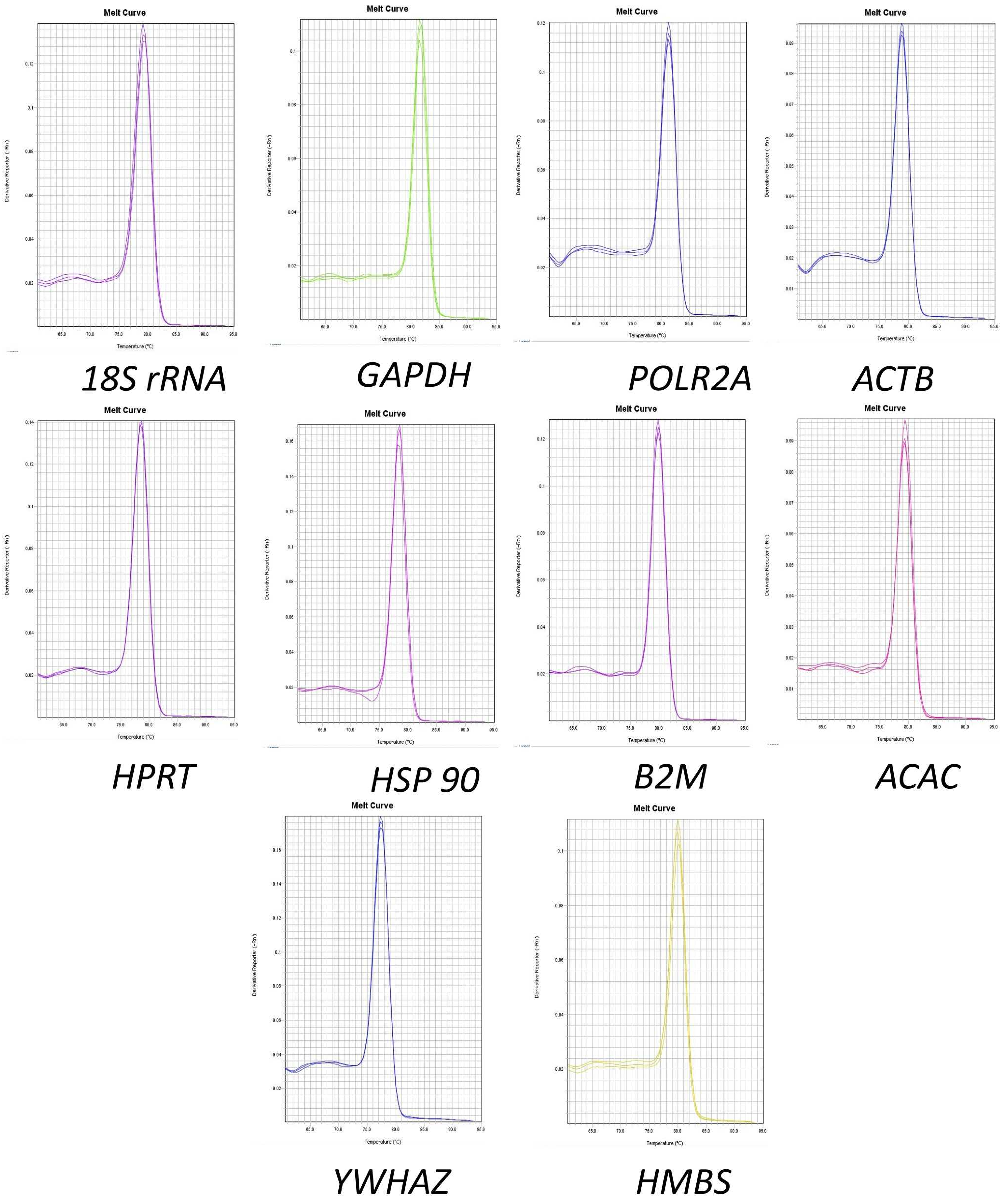
Melt Curve
Derivative Reporter (−Rn)
Temperature (°C)
18S rRNA
GAPDH
POLR2A
ACTB
HPRT
HSP 90
B2M
ACAC
YWHAZ
HMBS

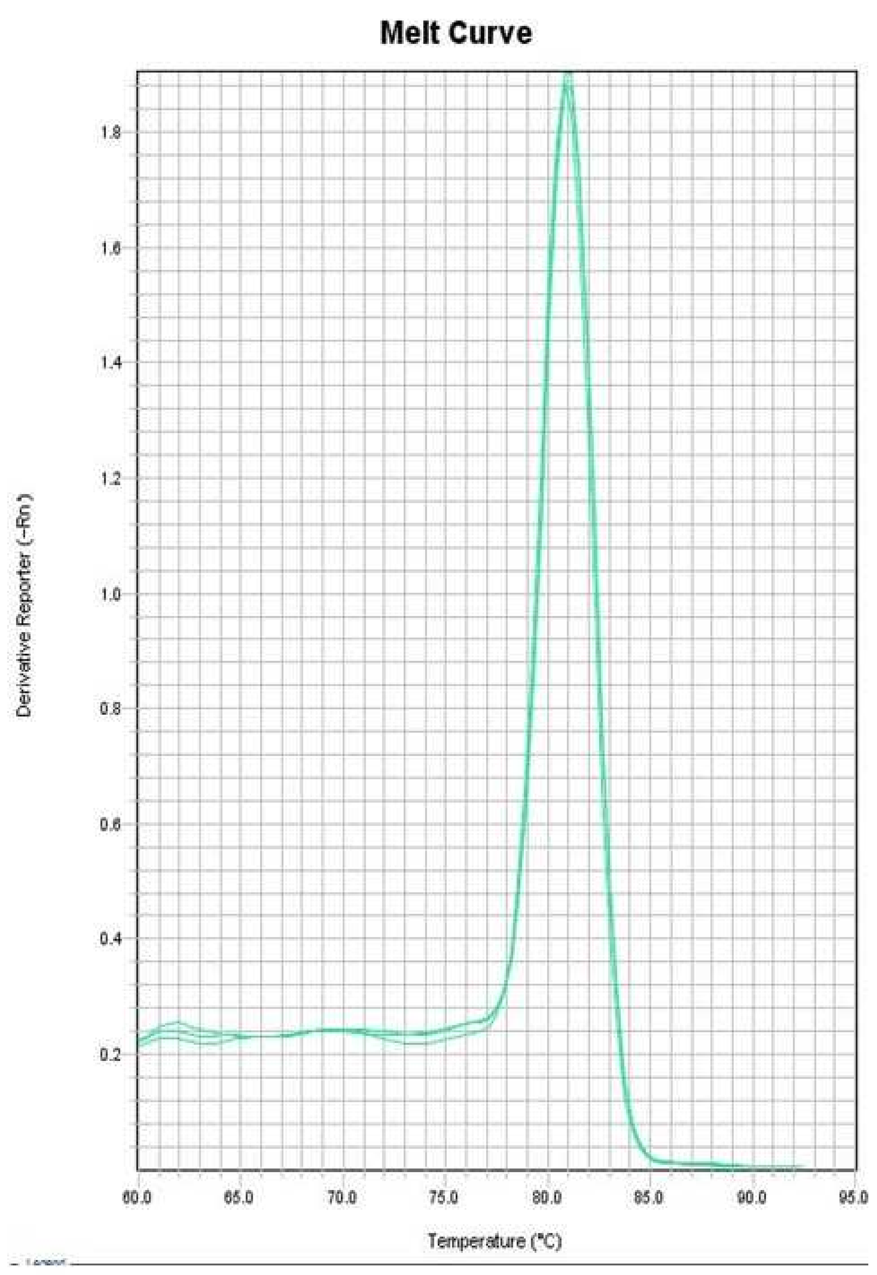

PPRV N

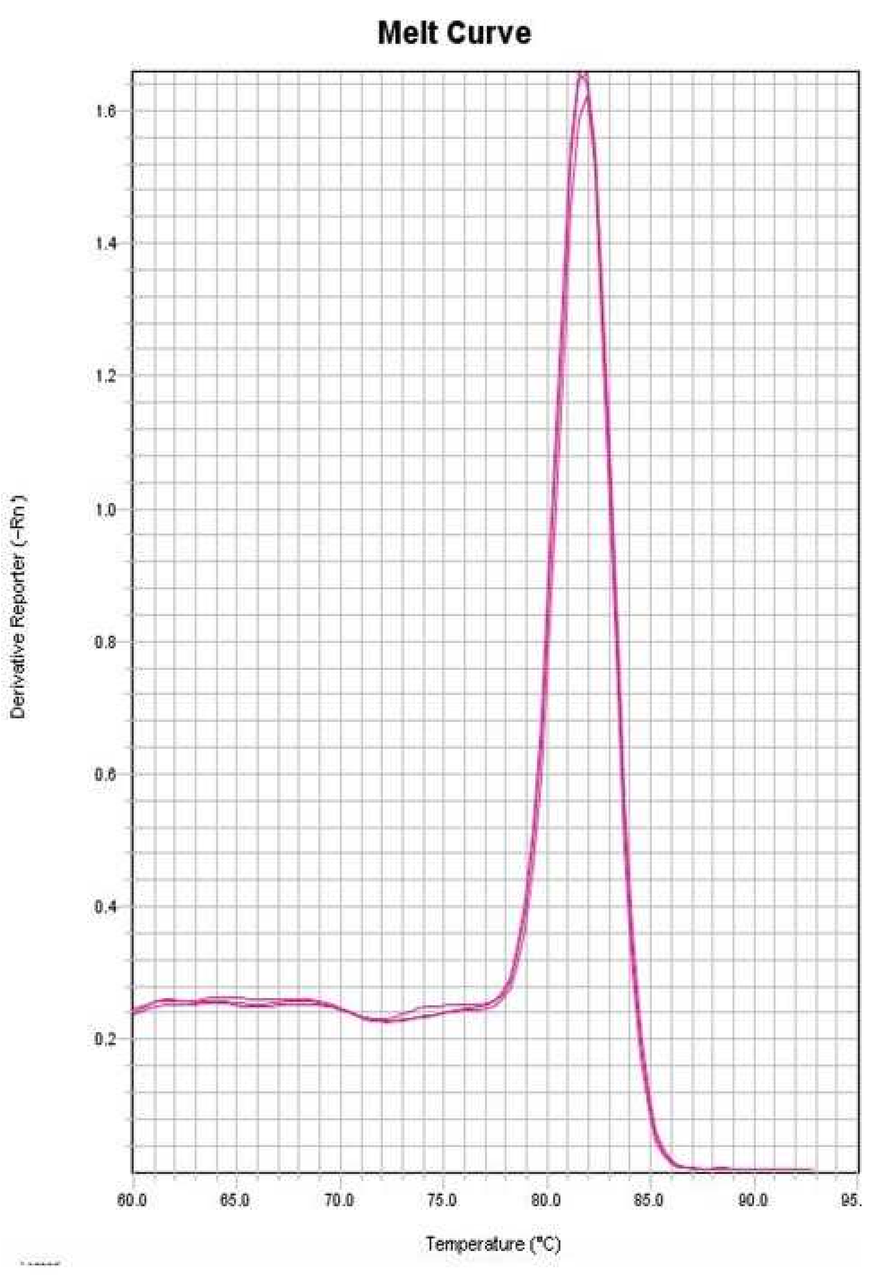

ISG15

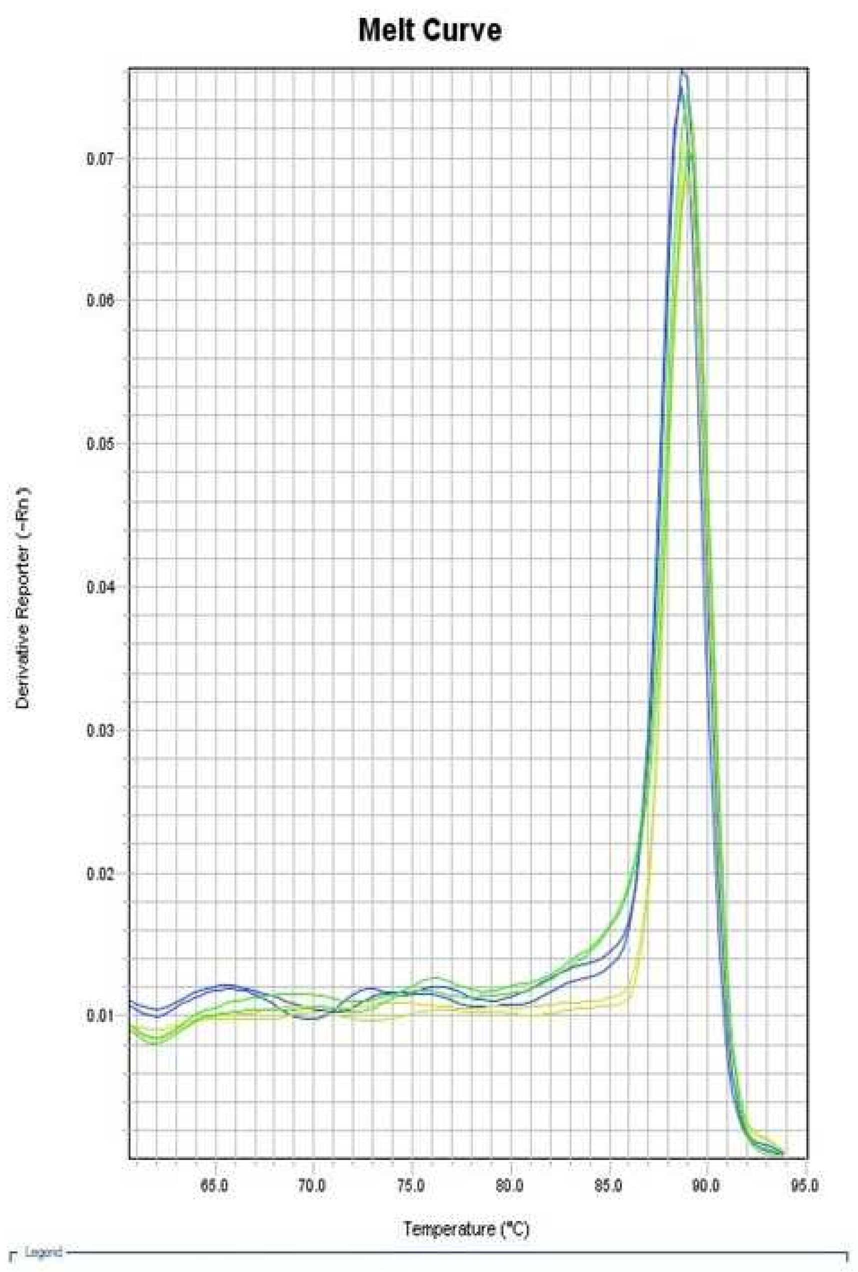

IRF7

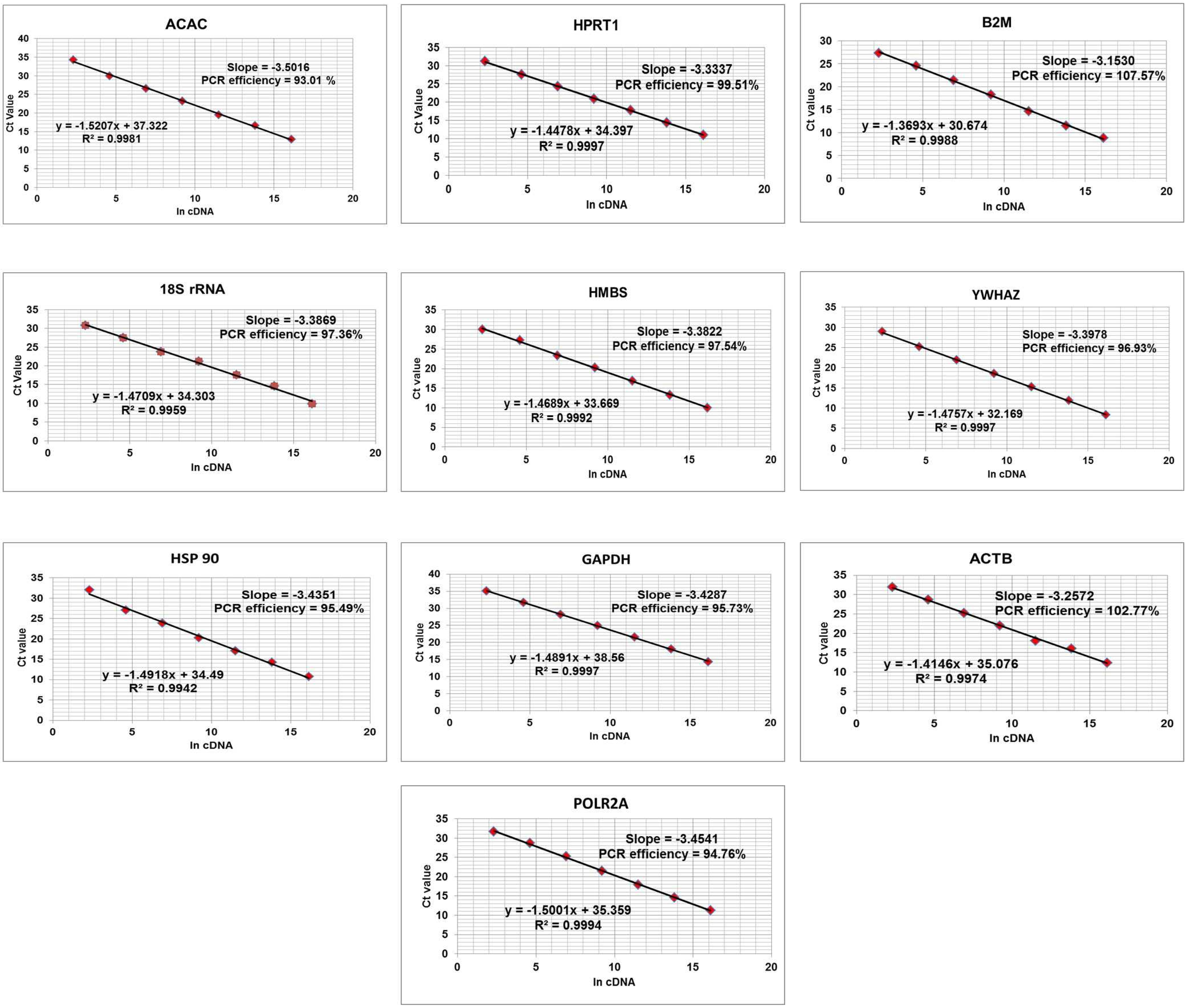
ACAC
Slope = -3.5016
PCR efficiency = 93.01 %
y = -1.5207x + 37.322
R² = 0.9981
Ct Value
ln cDNA
HPRT1
Slope = -3.3337
PCR efficiency = 99.51%
y = -1.4478x + 34.397
R² = 0.9997
Ct Value
ln cDNA
B2M
Slope = -3.1530
PCR efficiency = 107.57%
y = -1.3693x + 30.674
R² = 0.9988
Ct value
ln cDNA
18S rRNA
Slope = -3.3869
PCR efficiency = 97.36%
y = -1.4709x + 34.303
R² = 0.9959
Ct Value
ln cDNA
HMBS
Slope = -3.3822
PCR efficiency = 97.54%
y = -1.4689x + 33.669
R² = 0.9992
Ct value
ln cDNA
YWHAZ
Slope = -3.3978
PCR efficiency = 96.93%
y = -1.4757x + 32.169
R² = 0.9997
Ct value
ln cDNA
HSP 90
Slope = -3.4351
PCR efficiency = 95.49%
y = -1.4918x + 34.49
R² = 0.9942
Ct value
ln cDNA
GAPDH
Slope = -3.4287
PCR efficiency = 95.73%
y = -1.4891x + 38.56
R² = 0.9997
Ct value
ln cDNA
ACTB
Slope = -3.2572
PCR efficiency = 102.77%
y = -1.4146x + 35.076
R² = 0.9974
Ct value
ln cDNA
POLR2A
Slope = -3.4541
PCR efficiency = 94.76%
y = -1.5001x + 35.359
R² = 0.9994
Ct value
ln cDNA

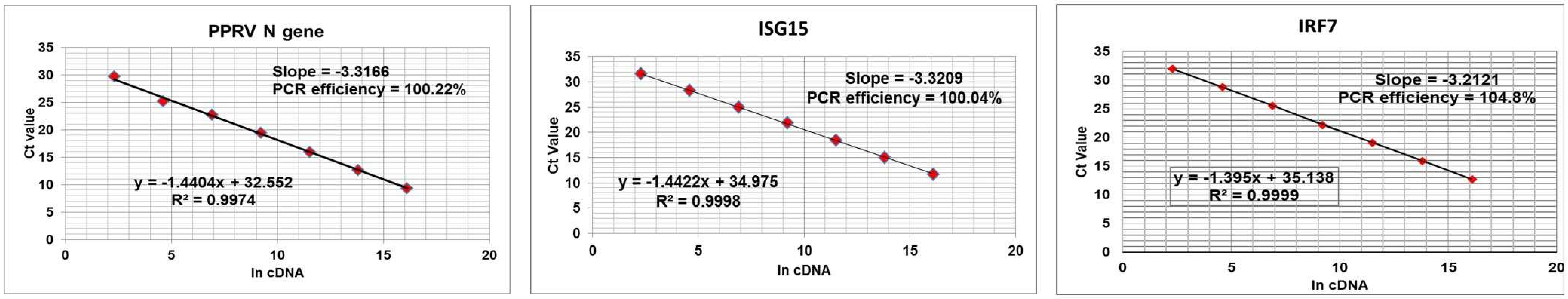

PPRV N gene
Slope = -3.3166
PCR efficiency = 100.22%
y = -1.4404x + 32.552
R² = 0.9974
Ct value
ln cDNA
ISG15
Slope = -3.3209
PCR efficiency = 100.04%
y = -1.4422x + 34.975
R² = 0.9998
Ct Value
ln cDNA
IRF7
Slope = -3.2121
PCR efficiency = 104.8%
y = -1.395x + 35.138
R² = 0.9999
Ct Value
ln cDNA

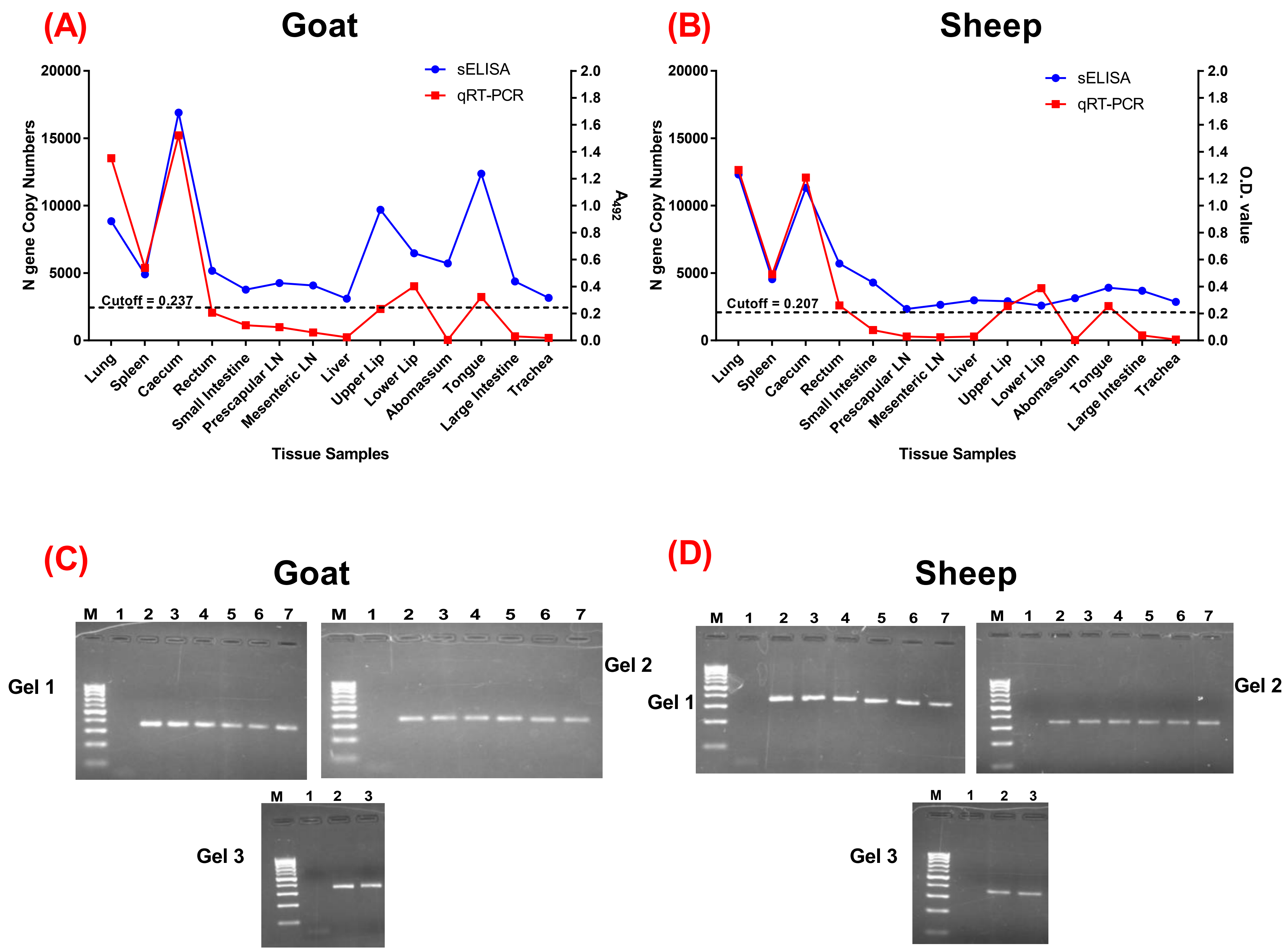

(A)
Goat
sELISA
qRT-PCR
N gene Copy Numbers
A492
Cutoff = 0.237
Lung
Spleen
Caecum
Rectum
Small Intestine
Prescapular LN
Mesenteric LN
Liver
Upper Lip
Lower Lip
Abomassum
Tongue
Large Intestine
Trachea
Tissue Samples
(B)
Sheep
sELISA
qRT-PCR
N gene Copy Numbers
O.D. value
Cutoff = 0.207
Tissue Samples
(C)
Goat
Gel 1
Gel 2
Gel 3
M 1 2 3 4 5 6 7
(D)
Sheep
Gel 1
Gel 2
Gel 3
M 1 2 3 4 5 6 7

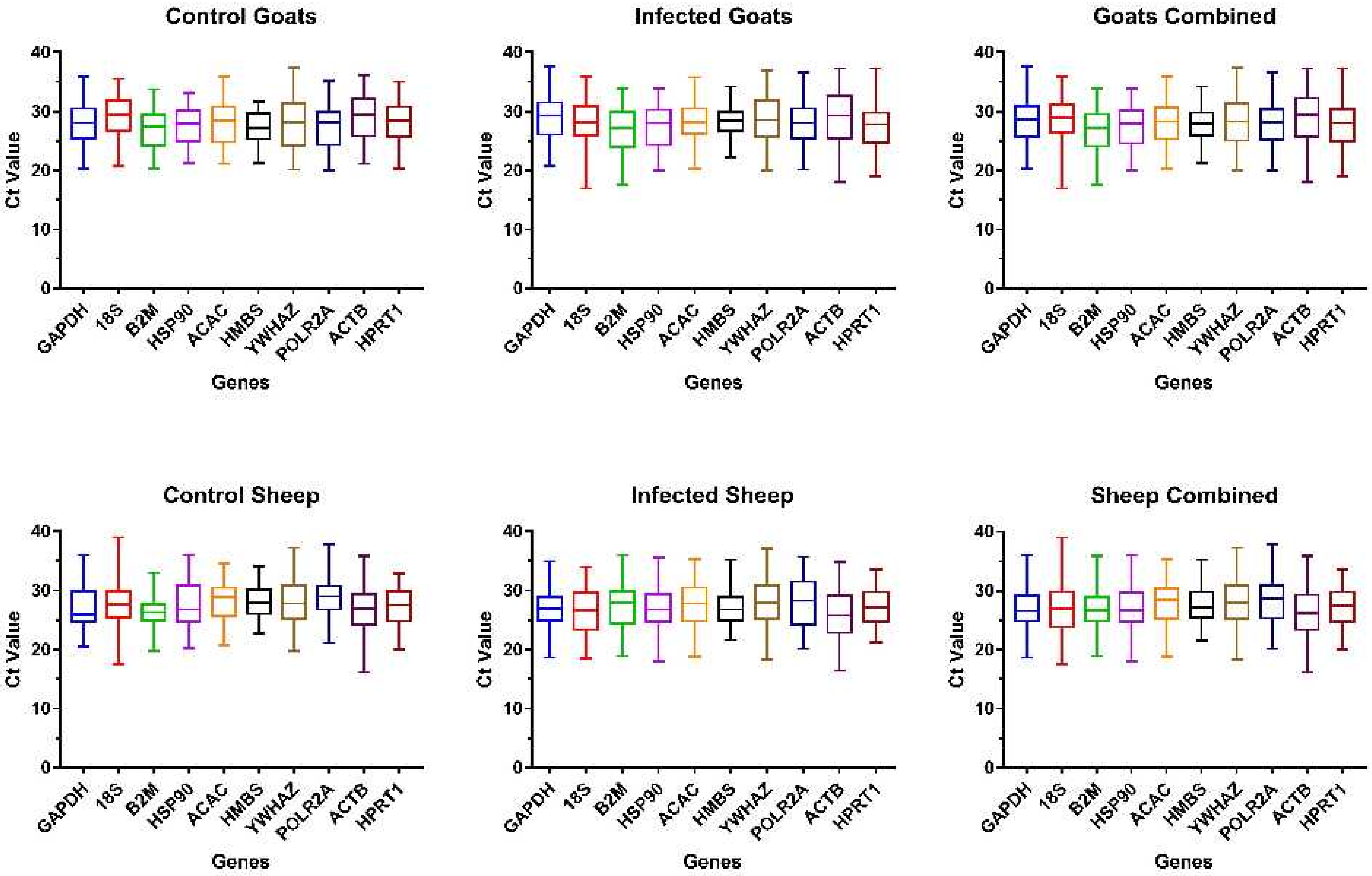
Control Goats
Infected Goats
Goats Combined
Control Sheep
Infected Sheep
Sheep Combined
Ct Value
0
10
20
30
40
GAPDH
18S
B2M
HSP90
ACAC
HMBS
YWHAZ
POLR2A
ACTB
HPRT1
Genes

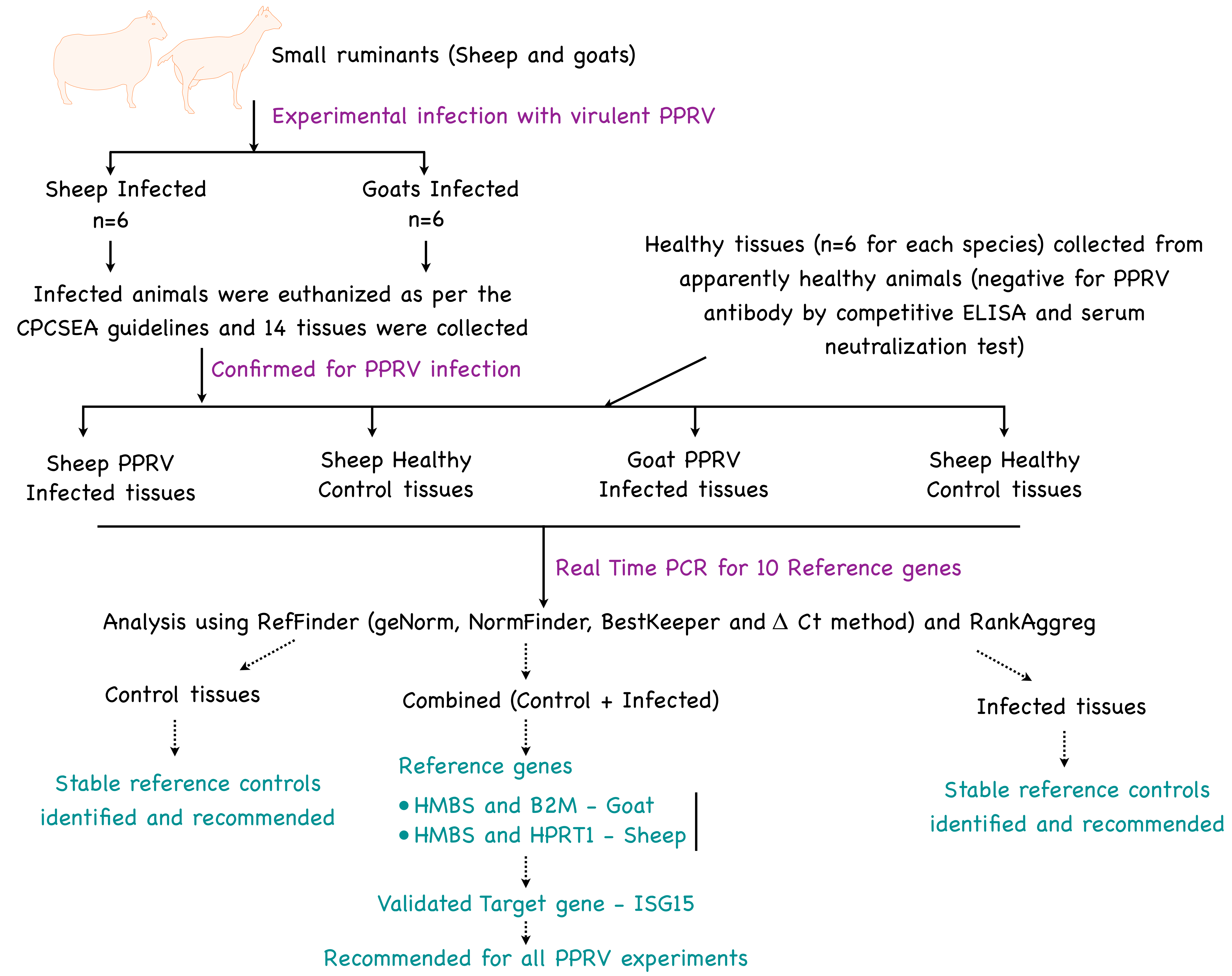
Small ruminants (Sheep and goats)
Experimental infection with virulent PPRV
Sheep Infected
n=6
Goats Infected
n=6
Infected animals were euthanized as per the CPCSEA guidelines and 14 tissues were collected
Healthy tissues (n=6 for each species) collected from apparently healthy animals (negative for PPRV antibody by competitive ELISA and serum neutralization test)
Confirmed for PPRV infection
Sheep PPRV Infected tissues
Sheep Healthy Control tissues
Goat PPRV Infected tissues
Sheep Healthy Control tissues
Real Time PCR for 10 Reference genes
Analysis using RefFinder (geNorm, NormFinder, BestKeeper and Δ Ct method) and RankAggreg
Control tissues
Combined (Control + Infected)
Infected tissues
Stable reference controls identified and recommended
Reference genes
• HMBS and B2M - Goat
• HMBS and HPRT1 - Sheep
Stable reference controls identified and recommended
Validated Target gene - ISG15
Recommended for all PPRV experiments

Supplementary Table Legends

Supplementary Table S1: Ct Value distribution for all the candidate reference gene in Goats and sheep across different groups

Supplementary Table S2: Stability Value and Ranking of Candidate Reference Genes by geNorm software in different groups in Goats and Sheep

Supplementary Table S3: Stability Value and Ranking of Candidate Reference Genes by NormFinder software in different groups in Goats and Sheep

Supplementary Table S4: Std dev [+/- CP] and Ranking of Candidate Reference Genes by BestKeeper software in different groups in Goats and Sheep

Supplementary Table S5: Average of STDEV and Ranking of Candidate Reference Genes by delta CT method in different groups in Goats and Sheep

Supplementary Table S6: Consensus Ranking of Candidate Reference Genes by RefFinder and RankAggreg method in different groups in Goats and Sheep

Supplementary Table S7: Consensus Ranking of Candidate Reference Genes by RankAggreg method in different tissues of Goats

Supplementary Table S8: Consensus Ranking of Candidate Reference Genes by RankAggreg method in different tissues of Sheep

Supplementary Table S9: MIQE checklist

Supplementary Table S1: Ct Value distribution w.r.t species and gene

Genes	Infected Goats Mean ± SEM	Control Goats Mean ± SEM	Goats Combined Mean ± SEM	Infected Sheep Mean ± SEM	Control Sheep Mean ± SEM	Sheep Combined Mean ± SEM
GAPDH	29.059±0.245	28.12±0.231	28.59±0.17	27.198±0.209	27.155±0.217	27.177±0.15
18S	28.074±0.246	29.12±0.216	28.597±0.165	26.458±0.234	27.391±0.237	26.924±0.168
B2M	26.891±0.228	26.988±0.204	26.939±0.153	27.474±0.248	26.339±0.171	26.907±0.153
HSP90	27.247±0.231	27.543±0.205	27.395±0.155	26.975±0.215	27.518±0.242	27.246±0.162
ACAC	28.157±0.224	28.176±0.243	28.166±0.165	27.604±0.251	28.338±0.203	27.971±0.162
HMBS	28.243±0.155	27.196±0.167	27.719±0.116	27.191±0.183	28.068±0.161	27.63±0.123
YWHAZ	28.404±0.246	28.037±0.258	28.221±0.178	27.916±0.257	28.105±0.228	28.01±0.172
POLR2A	28.008±0.227	27.539±0.234	27.773±0.163	27.939±0.264	28.726±0.2	28.332±0.166
ACTB	29.009±0.288	28.867±0.25	28.938±0.191	25.859±0.276	26.819±0.258	26.339±0.19

Supplementary Table S2: Stability Value and Ranking of Candidate Reference Genes by **geNorm** software in different groups in Goats and Sheep

Rank	1	2	3	4	5	6	7	8	9
**A. Control Goats**									
Gene Name	*B2M* | *HSP90*	*ACAC*	*HMBS*	*18S*	*HPRT1*	*GAPDH*	*POLR2A*	*ACTB*	*YWHAZ*
Stability Value	3.961	4.443	4.505	4.599	4.684	4.773	4.836	4.891	4.927
**B. Infected Goats**									
Gene Name	*GAPDH* | *HMBS*	*ACAC*	*POLR2A*	*B2M*	*HPRT1*	*18S*	*HSP90*	*YWHAZ*	*ACTB*
Stability Value	4.066	4.31	4.455	4.549	4.655	4.754	4.845	4.956	5.088
**C. Goats Combined**									
Gene Name	*ACAC* | *HMBS*	*B2M*	*HSP90*	*GAPDH*	*POLR2A*	*HPRT1*	*18S*	*YWHAZ*	*ACTB*
Stability Value	4.178	4.466	4.544	4.639	4.733	4.818	4.87	4.951	5.032
**D. Control Sheep**									
Gene Name	*HMBS* | *HPRT1*	*B2M*	*GAPDH*	*YWHAZ*	*POLR2A*	*ACAC*	*18S*	*HSP90*	*ACTB*
Stability Value	3.181	3.457	3.647	3.825	3.977	4.092	4.221	4.308	4.387
**E. Infected Sheep**									
Gene Name	*HMBS* | *HPRT1*	*GAPDH*	*18S*	*HSP90*	*B2M*	*ACAC*	*YWHAZ*	*POLR2A*	*ACTB*
Stability Value	3.858	4.212	4.329	4.457	4.532	4.608	4.662	4.747	4.83
**F. Sheep Combined**									
Gene Name	*HMBS* | *HPRT1*	*GAPDH*	*YWHAZ*	*B2M*	*18S*	*ACAC*	*HSP90*	*POLR2A*	*ACTB*
Stability Value	3.551	3.966	4.16	4.292	4.382	4.451	4.499	4.559	4.64

NB: lower the M value higher the stability

Supplementary Table S3: Stability Value and Ranking of Candidate Reference Genes by **NormFinder** software in different groups in Goats and Sheep

Rank	1	2	3	4	5	6	7	8	9	10
**A. Control Goats**										
Gene Name	*HMBS*	*HSP90*	*B2M*	*18S*	*HPRT1*	*ACAC*	*GAPDH*	*POLR2A*	*ACTB*	*YWHAZ*
Stability Value	2.896	3.273	3.364	3.421	3.537	3.65	3.652	3.666	3.694	3.707
**B. Infected Goats**										
Gene Name	*HMBS*	*ACAC*	*HPRT1*	*B2M*	*POLR2A*	*GAPDH*	*18S*	*HSP90*	*YWHAZ*	*ACTB*
Stability Value	2.084	3.238	3.488	3.531	3.559	3.56	3.93	3.944	3.999	4.413
**C. Goats Combined**										
Gene Name	*HMBS*	*B2M*	*ACAC*	*HPRT1*	*POLR2A*	*HSP90*	*GAPDH*	*18S*	*YWHAZ*	*ACTB*
Stability Value	2.576	3.444	3.445	3.536	3.614	3.625	3.633	3.732	3.854	4.064
**D. Control Sheep**										
Gene Name	*HMBS*	*B2M*	*GAPDH*	*HPRT1*	*YWHAZ*	*ACAC*	*18S*	*POLR2A*	*HSP90*	*ACTB*
Stability Value	2.354	2.623	3.005	3.05	3.081	3.213	3.279	3.286	3.492	3.6
**E. Infected Sheep**										
Gene Name	*HSP90*	*GAPDH*	*18S*	*HPRT1*	*HMBS*	*B2M*	*ACAC*	*YWHAZ*	*POLR2A*	*ACTB*
Stability Value	3.067	3.1	3.168	3.241	3.276	3.455	3.493	3.628	3.775	3.954
**F. Sheep Combined**										
Gene Name	*HMBS*	*GAPDH*	*HPRT1*	*B2M*	*18S*	*HSP90*	*ACAC*	*YWHAZ*	*POLR2A*	*ACTB*
Stability Value	2.86	3.058	3.145	3.18	3.233	3.282	3.356	3.362	3.54	3.789

Supplementary Table S4: Std dev [+/- CP] and Ranking of Candidate Reference Genes by **BestKeeper** software in different groups in Goats and Sheep

Rank	1	2	3	4	5	6	7	8	9	10
**A. Control Goats**										
Gene Name	*HMBS*	*B2M*	*18S*	*HSP90*	*HPRT1*	*GAPDH*	*POLR2A*	*ACAC*	*ACTB*	*YWHAZ*
Std dev [+/- CP]	2.21	2.77	2.8	2.8	2.89	2.94	3.08	3.25	3.38	3.47
**B. Infected Goats**										
Gene Name	*HMBS*	*ACAC*	*HPRT1*	*POLR2A*	*18S*	*B2M*	*GAPDH*	*HSP90*	*YWHAZ*	*ACTB*
Std dev [+/- CP]	1.95	2.8	2.9	2.94	3.01	3.06	3.14	3.15	3.23	3.77
**C. Goats Combined**										
Gene Name	*HMBS*	*HPRT1*	*B2M*	*18S*	*HSP90*	*POLR2A*	*ACAC*	*GAPDH*	*YWHAZ*	*ACTB*
Std dev [+/- CP]	2.14	2.91	2.92	2.96	2.97	3.01	3.02	3.08	3.35	3.57
**D. Control Sheep**										
Gene Name	*B2M*	*HMBS*	*POLR2A*	*HPRT1*	*ACAC*	*GAPDH*	*18S*	*YWHAZ*	*HSP90*	*ACTB*
Std dev [+/- CP]	2.02	2.12	2.46	2.65	2.66	2.83	2.97	3.06	3.19	3.33
**E. Infected Sheep**										
Gene Name	*HMBS*	*HPRT1*	*GAPDH*	*HSP90*	*18S*	*B2M*	*ACAC*	*YWHAZ*	*ACTB*	*POLR2A*
Std dev [+/- CP]	2.35	2.54	2.66	2.78	3.18	3.2	3.27	3.31	3.6	3.62
**F. Sheep Combined**										
Gene Name	*HMBS*	*HPRT1*	*B2M*	*GAPDH*	*HSP90*	*ACAC*	*POLR2A*	*18S*	*YWHAZ*	*ACTB*
Std dev [+/- CP]	2.28	2.6	2.69	2.75	2.98	2.98	3.07	3.09	3.18	3.49

Supplementary Table S5: Average of STDEV and Ranking of Candidate Reference Genes by **delta C_T** method in different groups in Goats and Sheep

Rank	1	2	3	4	5	6	7	8	9	10
**A. Control Goats**										
Gene Name	*HMBS*	*HSP90*	*B2M*	*18S*	*HPRT1*	*ACAC*	*GAPDH*	*POLR2A*	*ACTB*	*YWHAZ*
Average of STDEV	4.58	4.79	4.84	4.89	4.96	5.02	5.03	5.04	5.06	5.07
**B. Infected Goats**										
Gene Name	*HMBS*	*ACAC*	*HPRT1*	*B2M*	*POLR2A*	*GAPDH*	*18S*	*HSP90*	*YWHAZ*	*ACTB*
Average of STDEV	4.28	4.87	5.01	5.03	5.06	5.06	5.29	5.3	5.35	5.61
**C. Goats Combined**										
Gene Name	*HMBS*	*B2M*	*ACAC*	*HPRT1*	*POLR2A*	*HSP90*	*GAPDH*	*18S*	*YWHAZ*	*ACTB*
Average of STDEV	4.47	4.95	4.96	5.02	5.06	5.07	5.08	5.14	5.22	5.36
**D. Control Sheep**										
Gene Name	*HMBS*	*B2M*	*GAPDH*	*HPRT1*	*YWHAZ*	*ACAC*	*18S*	*POLR2A*	*HSP90*	*ACTB*
Average of STDEV	3.94	4.1	4.31	4.33	4.38	4.46	4.5	4.5	4.65	4.71
**E. Infected Sheep**										
Gene Name	*HSP90*	*GAPDH*	*18S*	*HPRT1*	*HMBS*	*B2M*	*ACAC*	*YWHAZ*	*POLR2A*	*ACTB*
Average of STDEV	4.62	4.64	4.68	4.71	4.72	4.85	4.88	4.96	5.07	5.16
**F. Sheep Combined**										
Gene Name	*HMBS*	*GAPDH*	*HPRT1*	*B2M*	*18S*	*HSP90*	*YWHAZ*	*ACAC*	*POLR2A*	*ACTB*
Average of STDEV	4.37	4.5	4.54	4.58	4.61	4.65	4.69	4.69	4.81	4.97

Supplementary Table S6: Consensus Ranking of Candidate Reference Genes by **RefFinder and RankAggreg** method in different groups in Goats and Sheep

Rank	1	2	3	4	5	6	7	8	9	10
**A. Control Goats**										
RefFinder	*HMBS*	*HSP90*	*B2M*	*18S*	*HPRT1*	*ACAC*	*GAPDH*	*POLR2A*	*ACTB*	*YWHAZ*
RankAggreg	*HMBS*	*HSP90*	*B2M*	*18S*	*ACAC*	*HPRT1*	*GAPDH*	*POLR2A*	*ACTB*	*YWHAZ*
**B. Infected Goats**										
RefFinder	*HMBS*	*ACAC*	*HPRT1*	*GAPDH*	*POLR2A*	*B2M*	*18S*	*HSP90*	*YWHAZ*	*ACTB*
RankAggreg	*HMBS*	*ACAC*	*GAPDH*	*HPRT1*	*POLR2A*	*B2M*	*18S*	*HSP90*	*YWHAZ*	*ACTB*
**C. Goats Combined**										
RefFinder	*HMBS*	*B2M*	*ACAC*	*HPRT1*	*HSP90*	*POLR2A*	*GAPDH*	*18S*	*YWHAZ*	*ACTB*
RankAggreg	*HMBS*	*B2M*	*ACAC*	*HPRT1*	*HSP90*	*POLR2A*	*GAPDH*	*18S*	*YWHAZ*	*ACTB*
**D. Control Sheep**										
RefFinder	*HMBS*	*B2M*	*HPRT1*	*GAPDH*	*YWHAZ*	*POLR2A*	*ACAC*	*18S*	*HSP90*	*ACTB*
RankAggreg	*HMBS*	*B2M*	*HPRT1*	*GAPDH*	*YWHAZ*	*ACAC*	*POLR2A*	*18S*	*HSP90*	*ACTB*
**E. Infected Sheep**										
RefFinder	*HSP90*	*HMBS*	*HPRT1*	*GAPDH*	*18S*	*B2M*	*ACAC*	*YWHAZ*	*POLR2A*	*ACTB*
RankAggreg	*HMBS*	*HSP90*	*HPRT1*	*GAPDH*	*18S*	*B2M*	*ACAC*	*YWHAZ*	*POLR2A*	*ACTB*
**F. Sheep Combined**										
RefFinder	*HMBS*	*HPRT1*	*GAPDH*	*B2M*	*18S*	*HSP90*	*YWHAZ*	*ACAC*	*POLR2A*	*ACTB*
RankAggreg	*HMBS*	*HPRT1*	*GAPDH*	*B2M*	*18S*	*YWHAZ*	*HSP90*	*ACAC*	*POLR2A*	*ACTB*

Supplementary Table S7: Consensus Ranking of Candidate Reference Genes by **RankAggreg** method in different tissues of Goats

Tissue Samples	1	2	3	4	5	6	7	8	9	10
Lung	*POLR2A*	*ACTB*	*HMBS*	*18S*	*ACAC*	*GAPDH*	*HSP90*	*YWHAZ*	*B2M*	*HPRT1*
Spleen	*HMBS*	*YWHAZ*	*B2M*	*18S*	*HSP90*	*ACAC*	*HPRT1*	*ACTB*	*GAPDH*	*POLR2A*
Caecum	*HMBS*	*B2M*	*YWHAZ*	*18S*	*HPRT1*	*ACAC*	*POLR2A*	*GAPDH*	*ACTB*	*HSP90*
Rectum	*GAPDH*	*HMBS*	*HPRT1*	*18S*	*B2M*	*ACAC*	*YWHAZ*	*POLR2A*	*HSP90*	*ACTB*
Small Intestine	*HMBS*	*ACAC*	*18S*	*ACTB*	*HSP90*	*B2M*	*HPRT1*	*GAPDH*	*YWHAZ*	*POLR2A*
Prescapular Lymphnode	*GAPDH*	*HMBS*	*POLR2A*	*HPRT1*	*HSP90*	*YWHAZ*	*18S*	*ACTB*	*ACAC*	*B2M*
Mesenteric Lymphnode	*GAPDH*	*HMBS*	*B2M*	*HPRT1*	*ACAC*	*18S*	*HSP90*	*YWHAZ*	*POLR2A*	*ACTB*
Liver	*POLR2A*	*HMBS*	*18S*	*HPRT1*	*ACAC*	*ACTB*	*B2M*	*HSP90*	*YWHAZ*	*GAPDH*
Upper Lip	*B2M*	*HMBS*	*HSP90*	*POLR2A*	*ACAC*	*18S*	*GAPDH*	*ACTB*	*HPRT1*	*YWHAZ*
Lower Lip	*HMBS*	*ACAC*	*B2M*	*ACTB*	*18S*	*GAPDH*	*HSP90*	*YWHAZ*	*POLR2A*	*HPRT1*
Abomassum	*GAPDH*	*HSP90*	*ACAC*	*HPRT1*	*HMBS*	*POLR2A*	*18S*	*B2M*	*ACTB*	*YWHAZ*
Tongue	*ACAC*	*POLR2A*	*HMBS*	*B2M*	*GAPDH*	*YWHAZ*	*HSP90*	*HPRT1*	*18S*	*ACTB*
Large Intestine	*HMBS*	*HPRT1*	*POLR2A*	*GAPDH*	*YWHAZ*	*18S*	*ACTB*	*HSP90*	*ACAC*	*B2M*
Trachea	*HMBS*	*POLR2A*	*B2M*	*18S*	*HPRT1*	*YWHAZ*	*ACAC*	*HSP90*	*GAPDH*	*ACTB*

Supplementary Table S8: Consensus Ranking of Candidate Reference Genes by **RankAggreg** method in different tissues of Sheep

Tissue Samples	1	2	3	4	5	6	7	8	9	10
Lung	*B2M*	*ACAC*	*HSP90*	*HPRT1*	*HMBS*	*POLR2A*	*YWHAZ*	*GAPDH*	*18S*	*ACTB*
Spleen	*ACTB*	*18S*	*HSP90*	*ACAC*	*GAPDH*	*B2M*	*POLR2A*	*HMBS*	*YWHAZ*	*HPRT1*
Caecum	*HMBS*	*GAPDH*	*ACAC*	*HPRT1*	*YWHAZ*	*18S*	*POLR2A*	*B2M*	*ACTB*	*HSP90*
Rectum	*B2M*	*HMBS*	*ACAC*	*POLR2A*	*HSP90*	*18S*	*ACTB*	*GAPDH*	*HPRT1*	*YWHAZ*
Small Intestine	*GAPDH*	*ACAC*	*YWHAZ*	*HPRT1*	*HMBS*	*B2M*	*HSP90*	*18S*	*ACTB*	*POLR2A*
Prescapular Lymphnode	*ACAC*	*B2M*	*GAPDH*	*HMBS*	*YWHAZ*	*HPRT1*	*HSP90*	*POLR2A*	*18S*	*ACTB*
Mesenteric Lymphnode	*HPRT1*	*HSP90*	*B2M*	*GAPDH*	*HMBS*	*ACAC*	*18S*	*YWHAZ*	*POLR2A*	*ACTB*
Liver	*ACTB*	*HPRT1*	*YWHAZ*	*ACAC*	*18S*	*GAPDH*	*HMBS*	*HSP90*	*POLR2A*	*B2M*
Upper Lip	*HSP90*	*HMBS*	*18S*	*B2M*	*YWHAZ*	*POLR2A*	*ACAC*	*HPRT1*	*GAPDH*	*ACTB*
Lower Lip	*HMBS*	*HPRT1*	*ACTB*	*GAPDH*	*YWHAZ*	*ACAC*	*HSP90*	*18S*	*B2M*	*POLR2A*
Abomassum	*HPRT1*	*GAPDH*	*HMBS*	*POLR2A*	*ACTB*	*HSP90*	*18S*	*B2M*	*YWHAZ*	*ACAC*
Tongue	*YWHAZ*	*B2M*	*18S*	*GAPDH*	*HMBS*	*HPRT1*	*HSP90*	*ACAC*	*ACTB*	*POLR2A*
Large Intestine	*GAPDH*	*POLR2A*	*18S*	*B2M*	*HPRT1*	*HMBS*	*ACTB*	*ACAC*	*YWHAZ*	*HSP90*
Trachea	*HMBS*	*POLR2A*	*HPRT1*	*GAPDH*	*ACAC*	*HSP90*	*YWHAZ*	*B2M*	*ACTB*	*18S*

Supplementary Table S9: MIQE Checklist

ITEM TO CHECK	IMPORTANCE	CHECKLIST	REMARKS
Definition of experimental and control groups	E	Y	
Number within each group	E	Y	
Assay carried out by core lab or investigator's lab?	D	Y	Investigator's Lab
Acknowledgement of authors' contributions	D	Y	
Description	E	Y	
Volume/mass of sample processed	D	Y	
Microdissection or macrodissection	E	Y	
Processing procedure	E	Y	
If frozen - how and how quickly?	E	NA	
If fixed - with what, how quickly?	E	NA	
Sample storage conditions and duration (especially for FFPE samples)	E	Y	
Procedure and/or instrumentation	E	Y	
Name of kit and details of any modifications	E	Y	
Source of additional reagents used	D	Y	
Details of DNase or RNAse treatment	E	Y	
Contamination assessment (DNA or RNA)	E	Y	
Nucleic acid quantification	E	Y	
Instrument and method	E	Y	
Purity (A260/A280)	D	Y	
Yield	D	Y	
RNA integrity method/instrument	E	Y	
RIN/RQI or Cq of 3' and 5' transcripts	E	Y	
Electrophoresis traces	D	NA	
Inhibition testing (Cq dilutions, spike or other)	E	Y	
Complete reaction conditions	E	Y	
Amount of RNA and reaction volume	E	Y	
Priming oligonucleotide (if using GSP) and concentration	E	Y	
Reverse transcriptase and concentration	E	Y	
Temperature and time	E	Y	
Manufacturer of reagents and catalogue numbers	D	Y	
Cqs with and without RT	D*	NA	
Storage conditions of cDNA	D	Y	
If multiplex, efficiency and LOD of each assay.	E	Y	
Sequence accession number	E	Y	
Location of amplicon	D	NA	
Amplicon length	E	Y	
In silico specificity screen (BLAST, etc)	E	Y	
Pseudogenes, retropseudogenes or other homologs?	D	NA	
Sequence alignment	D	NA	
Secondary structure analysis of amplicon	D	NA	
Location of each primer by exon or intron (if applicable)	E	NA	
What splice variants are targeted?	E	NA	
Primer sequences	E	Y	
RTPrimerDB Identification Number	D	NA	
Probe sequences	D**	NA	
Location and identity of any modifications	E	NA	
Manufacturer of oligonucleotides	D	NA	
Purification method	D	NA	
Complete reaction conditions	E	Y	
Reaction volume and amount of cDNA/DNA	E	Y	
Primer, (probe), Mg++ and dNTP concentrations	E	Y	
Polymerase identity and concentration	E	Y	
Buffer/kit identity and manufacturer	E	Y	
Exact chemical constitution of the buffer	D	NA	
Additives (SYBR Green I, DMSO, etc.)	E	Y	
Manufacturer of plates/tubes and catalog number	D	Y	
Complete thermocycling parameters	E	Y	
Reaction setup (manual/robotic)	D	Y	Manual
Manufacturer of qPCR instrument	E	Y	Applied Biosystem
Evidence of optimisation (from gradients)	D	NA	
Specificity (gel, sequence, melt, or digest)	E	Y	Melt Curve Analysis
For SYBR Green I, Cq of the NTC	E	Y	Undetermined for all NTC wells
Standard curves with slope and y-intercept	E	Y	using 7 point, 10 fold serial dilution with triplicate
PCR efficiency calculated from slope	E	Y	Figure S1
Confidence interval for PCR efficiency or standard error	D	NA	
r2 of standard curve	E	Y	Figure S1
Linear dynamic range	E	Y	
Cq variation at lower limit	E	Y	
Confidence intervals throughout range	D	NA	
Evidence for limit of detection	E		Ct < 40 for all reactions
If multiplex, efficiency and LOD of each assay.	E	NA	
qPCR analysis program (source, version)	E	Y	
Cq method determination	E	Y	
Outlier identification and disposition	E	Y	
Results of NTCs	E	Y	Ct value undermined in all NTC wells
Justification of number and choice of reference genes	E	Y	
Description of normalisation method	E	Y	
Number and concordance of biological replicates	D	Y	
Number and stage (RT or qPCR) of technical replicates	E	Y	
Repeatability (intra-assay variation)	E	Y	
Reproducibility (inter-assay variation, %CV)	D	NA	
Power analysis	D	NA	
Statistical methods for result significance	E	Y	
Software (source, version)	E	Y	
Cq or raw data submission using RDML	D	NA	

Table 1. MIQE checklist for authors, reviewers and editors. All essential information (E) must be submitted with the manuscript. Desirable information (D) should be submitted if available. If using primers obtained from RTPrimerDB, information on qPCR target, oligonucleotides, protocols and validation is available from that source.

*: Assessing the absence of DNA using a no RT assay is essential when first extracting RNA. Once the sample has been validated as RDNA-free, inclusion of a no-RT control is desirable, but no longer essential.

**: Disclosure of the probe sequence is highly desirable and strongly encouraged. However, since not all commercial pre-designed assay vendors provide this information, it cannot be an essential requirement. Use of such assays is advised against.
